# Unravelling the molecular control of calvarial suture fusion in children with craniosynostosis

**DOI:** 10.1186/1471-2164-8-458

**Published:** 2007-12-12

**Authors:** Anna K Coussens, Christopher R Wilkinson, Ian P Hughes, C Phillip Morris, Angela van Daal, Peter J Anderson, Barry C Powell

**Affiliations:** 1 Cooperative Research Centre for Diagnostics, Institute of Health and Biomedical Innovation, Queensland University of Technology, Brisbane 4001, Australia; 2 Women's and Children's Health Research Institute, Adelaide 5006, Australia; 3 Faculty of Engineering, Computer & Mathematical Sciences, University of Adelaide, Adelaide 5005, Australia; 4 Faculty of Health Sciences, University of Adelaide, Adelaide 5005, Australia; 5 Australian Craniofacial Unit, Adelaide 5006, Australia; 6 Faculty of Health Sciences and Medicine, Bond University, Gold Coast 4229, Australia

## Abstract

**Background:**

Craniosynostosis, the premature fusion of calvarial sutures, is a common craniofacial abnormality. Causative mutations in more than 10 genes have been identified, involving fibroblast growth factor, transforming growth factor beta, and Eph/ephrin signalling pathways. Mutations affect each human calvarial suture (coronal, sagittal, metopic, and lambdoid) differently, suggesting different gene expression patterns exist in each human suture. To better understand the molecular control of human suture morphogenesis we used microarray analysis to identify genes differentially expressed during suture fusion in children with craniosynostosis. Expression differences were also analysed between each unfused suture type, between sutures from syndromic and non-syndromic craniosynostosis patients, and between unfused sutures from individuals with and without craniosynostosis.

**Results:**

We identified genes with increased expression in unfused sutures compared to fusing/fused sutures that may be pivotal to the maintenance of suture patency or in controlling early osteoblast differentiation (i.e. *RBP4*, *GPC3*, *C1QTNF3*, *IL11RA*, *PTN*, *POSTN*). In addition, we have identified genes with increased expression in fusing/fused suture tissue that we suggest could have a role in premature suture fusion (i.e. *WIF1*, *ANXA3*, *CYFIP2*). Proteins of two of these genes, glypican 3 and retinol binding protein 4, were investigated by immunohistochemistry and localised to the suture mesenchyme and osteogenic fronts of developing human calvaria, respectively, suggesting novel roles for these proteins in the maintenance of suture patency or in controlling early osteoblast differentiation. We show that there is limited difference in whole genome expression between sutures isolated from patients with syndromic and non-syndromic craniosynostosis and confirmed this by quantitative RT-PCR. Furthermore, distinct expression profiles for each unfused suture type were noted, with the metopic suture being most disparate. Finally, although calvarial bones are generally thought to grow without a cartilage precursor, we show histologically and by identification of cartilage-specific gene expression that cartilage may be involved in the morphogenesis of lambdoid and posterior sagittal sutures.

**Conclusion:**

This study has provided further insight into the complex signalling network which controls human calvarial suture morphogenesis and craniosynostosis. Identified genes are candidates for targeted therapeutic development and to screen for craniosynostosis-causing mutations.

## Background

Calvarial bones form by the proliferation and differentiation of multipotent mesenchymal cells into osteoblasts. This process, known as intramembranous ossification, is distinct from the development of the majority of other bones in the body which form by the ossification of a pre-existing cartilaginous matrix (endochondral ossification). Calvaria first form from a condensation of mesenchyme termed the primary centre of ossification. Mesenchymal cell proliferation and subsequent differentiation into osteoblasts occurs at the margins and the bone grows in a radial fashion until the osteogenic fronts of two calvaria approximate each other and structures called sutures form between the bones [1]. These intervening fibrous sutures act as flexible joints between the developing bones allowing the skull to change shape and grow during development. Maintenance of growth at the osteogenic fronts at the edges of the sutures requires a fine balance between proliferation and differentiation. Additionally, apoptosis has a role ensuring that the two osteogenic fronts remain separated [2]. Disruption of any of these processes can result in the premature fusion of calvarial sutures, known as craniosynostosis.

Craniosynostosis is amongst the most common cranial defects, second only to cleft palate. It occurs in 1 in 2500 live births and can be associated with significant morbidity, including mental retardation, deafness, and blindness, in addition to the significant social stigma associated with craniofacial deformation [3]. The condition may be caused by various genetic mutations, exposure to teratogens such as retinoic acid, mechanical stress, or result from certain metabolic or haematologic disorders [4,5]. Non-syndromic craniosynostosis refers to sporadic suture fusion in the absence of other developmental abnormalities and most commonly affects the sagittal suture. Syndromic craniosynostosis occurs as a result of simple genetic mutations and is accompanied by additional developmental abnormalities particularly involving the limbs [6]. Syndromic forms of craniosynostosis commonly affect the coronal suture but other sutures may be affected depending on the underlying genetic mutation. *FGFR2 *mutations are the most common and most severe affecting the coronal, metopic, sagittal, and lambdoid sutures. *FGFR3 *mutations affect the coronal and/or metopic sutures. *FGFR1*, *TWIST1 *and *EFNB1 *mutations generally affect only the coronal suture. *FNB1 *and *TGFBR1 *mutations have been associated with synostosis of the sagittal and/or lambdoid sutures, while gain-of-function *MSX2 *mutations result in synostosis of the coronal and sagittal sutures (reviewed in [7]).

The large number of genes identified as causal for craniosynostosis suggests that a complex molecular network controls suture morphogenesis in humans. In addition, rodent studies have revealed a role in suture formation for transforming growth factor beta (TGFβ) signalling mediated by various bone morphogenetic proteins (BMPs) [8-11]. Targeted functional genetic approaches are slowly unravelling the molecular signalling that controls suture morphogenesis. However, there is also a need for a broad experimental approach aimed at identifying all genes and, subsequently, their associated pathways which are essential to suture morphogenesis.

The different phenotypes induced by the known mutations suggest that distinct molecular pathways may be operating in different sutures. This is particularly evident in the case of the metopic suture which, in humans, normally fuses shortly after birth, while the other sutures remain patent until adulthood. This feature of the metopic suture may be explained by the finding in rodents that the frontal suture (equivalent to the metopic suture in humans) is populated by neural crest derived mesenchyme and separates the frontal bones, also of neural crest origin, while the other sutures are a juxtaposition of neural crest and paraxial mesoderm [12-14]. To understand the mechanisms of the fusion process gene expression profiles between the fusing posterior frontal sutures in mice have been compared to profiles from unfused sagittal and coronal sutures [15-20]. However, given that the signalling pathways controlling suture fusion are likely to differ in sutures derived from different developmental origins, it is unclear what such comparisons tell us about these fusion processes. There is, therefore, a need to study differential gene expression between fused and unfused sutures of the same developmental origin.

Subtle differences in cranial biology also exist between rodents and humans. For example, the rodent model created for the Pro250Arg *FGFR1 *mutation, which causes Pfeiffer syndrome, develops synostosis of the frontal, sagittal and coronal sutures [21] whereas in humans this mutation commonly affects only the coronal suture. Furthermore, primary cells cultured from patients with *FGFR2 *mutations and mice generated with the same mutations show differing proliferation and differentiation characteristics (reviewed in [22]). These differences emphasise that mechanisms controlling rodent suture morphogenesis do not exactly mimic those occurring in human sutures.

In this study we have analysed global *in vivo *expression differences between fused, fusing, and unfused sutures from patients with craniosynostosis to identify genes which are involved in maintaining suture patency and driving suture fusion within each human suture.

## Results

**Figure 1:**
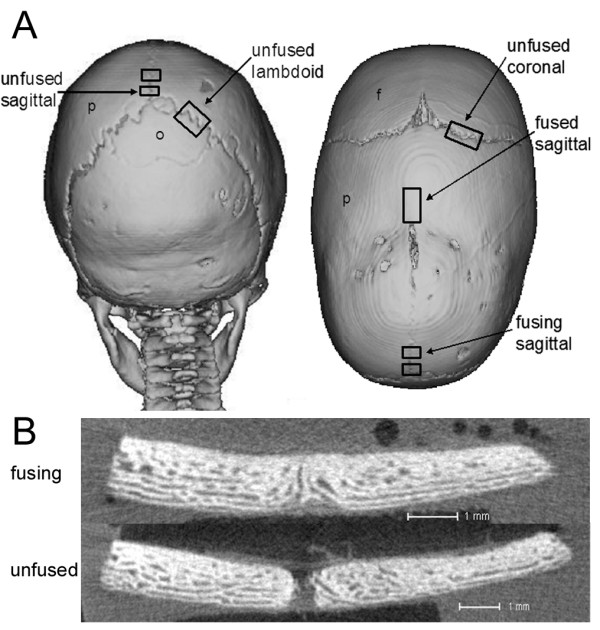
**Computer tomography (CT) scans showing site and fusion state of sutures obtained from craniosynostosis patients**. **A) **Posterior and superior (left and right) view of patient #58 indicating where unfused, fusing and fused sutures were obtained from. p, parietal bone; o, occipital bone; f, frontal bone. **B) **MicroCT image demonstrating a fusing and unfused suture. Scale 1 mm.

Five patients were recruited to the gene identification stage of this study, one diagnosed with syndromic craniosynostosis (Apert syndrome [MIM 101200]) and four with non-syndromic craniosynostosis (Table 1). Sixteen suture samples were obtained from these patients for microarray analysis; nine from unfused sutures, two from fusing, and five from fused sutures (Fig. 1A). To minimise any age-related changes and to eliminate any sex-related effects on the development of craniosynostosis, sutures were obtained from males aged 3-7 months. The stage of fusion was confirmed by 3D computer tomography (CT), MicroCT, and histological analysis and classified as unfused, fusing, or fused (Fig. 1). We performed microarray analyses on RNA isolated from sutures resected at surgery using the Affymetrix Human U133A 2.0 GeneChip platform containing ~18,000 gene transcripts. Microarray data were initially assessed using a number of quality control measures (Additional files 1, 2, 3). RNA digestion plots indicated that all samples showed a high similarity in RNA quality except for one unfused sagittal sample from patient #36 (US36). NUSE and Mbox plots indicated that this sample had a similar expression intensity compared to other samples but had elevated standard errors. We were initially cautious in our interpretation of any particular difference in expression seen with this sample.

**Table 1:** Phenotypes of patients, identified causative mutations, fusion state and site of obtained sutures.

**Analysis**	**Patient**	**Phenotype**	**Sex**	**Age (m)**	**Mutation**	**Fused Suture**	**Fusing Suture**	**Unfused Suture**
*Microarray*	#42	Apert syndrome	M	7	FGFR2 Ser252Trp	Coronal^a^ Lambdoid^a^	Metopic^a^	
	#36	Unicoronal synostosis	M	3	No FGFR or TWIST1		Coronal^a^	Coronal^a^ Sagittal
	#46	Sagittal synostosis	M	5	No FGFR or TWIST1	Sagittal		Coronal Lambdoid^a^ Metopic^a^
	#50	Sagittal synostosis	M	6	No FGFR or TWIST1	Sagittal		Coronal
	#58	Sagittal synostosis	M	7	No FGFR or TWIST1	Sagittal^a^	Sagittal^b^	Sagittal^a^ Coronal Lambdoid^a^
*Validation*	#47	Apert syndrome	F	38	FGFR2 Pro253Arg	Metopic		
	#85	Apert syndrome	F	40	FGFR2 Ser252Trp	Sagittal	Metopic	
	#90	Apert syndrome	F	4	FGFR2 Pro253Arg	Coronal	Coronal^c^	Sagittal Metopic
	#61	Apert syndrome	M	20	FGFR2 Ser252Trp		Coronal	Coronal
	#92	Muenke syndrome	F	6	FGFR3 Pro250Arg			Coronal
	#104	Saethre-Chotzen syndrome	M	14	TWIST1 c.256_276dup	Sagittal Metopic		
	#49	Metopic synostosis	M	10	No FGFR or TWIST1	Metopic		
	#60	Sagittal synostosis	F	9	No FGFR or TWIST1	Sagittal	Sagittal	
	#63	Sagittal synostosis	M	10	No FGFR or TWIST1	Sagittal	Sagittal	Lambdoid
	#72	Lambdoid synostosis	M	26	No FGFR or TWIST1	Lambdoid		
	#91	Sagittal synostosis	F	5	No FGFR or TWIST1			Metopic
	#94	Metopic synostosis	F	9	No FGFR or TWIST1	Metopic	Metopic	Sagittal
	#95	Unicoronal synostosis	M	5	No FGFR or TWIST1	Coronal		
	#41	Normal – Tessier Cleft	F	91	Not tested			Metopic
	#69	Normal – hydrocephalus	F	4	Not tested			Lambdoid
	#73	Normal – cerebellar tumour	M	9	Not tested			Coronal
	#81	Normal – hydrocephalus	F	2	Not tested			Lambdoid
	#87	Normal – cerebellar tumour	M	57	Not tested			Coronal
	#89	Normal – hydrocephalus	M	1 day	Not tested			Lambdoid
*Histology*	#3	Apert syndrome	M	4	FGFR2 Ser252Trp	Lambdoid		
	#5	Sagittal synostosis	M	3	No FGFR or TWIST1	Sagittal		
	#80	Metopic synostosis	M	6	No FGFR or TWIST1			Coronal Sagittal
	#83	Sagittal synostosis	M	6	No FGFR or TWIST1			Lambdoid (x2) Coronal
	#84	Unicoronal synostosis	F	7	No FGFR or TWIST1	Coronal		Sagittal

^a^RNA sample also used for qRT-PCR validation experiments.

^b^Sample used for validation experiments, but not microarray analysis.

^c^Two samples of varying degrees of fusion were obtained. The sample which was at the earlier stage of fusion was used for validation qRT-PCR; the sample at a later stage of fusion was used for within patient comparison only.

### Patient genetic background does not adversely affect gene expression

Hierarchical clustering, based on whole genome expression, showed samples typically grouped according to stage of fusion or suture type, and not solely by patient of origin, indicating no adverse patient-specific genetic background biases existed (Additional file 4). Importantly, sutures from the Apert syndrome patient grouped more closely with similar sutures from other non-syndromic patients, than they did to each other. These similarities in gene expression between syndromic and non-syndromic patients were confirmed with additional syndromic samples using realtime quantitative RT-PCR (qRT-PCR), as described later. This indicates that patient genetic background does not overly impact on gene expression and it provides proof of principle that the combined analysis of syndromic and non-syndromic patient samples can be applied in the study of general mechanisms of craniosynostosis.

### Metopic sutures have different gene expression profiles to other sutures

**Figure 2:**
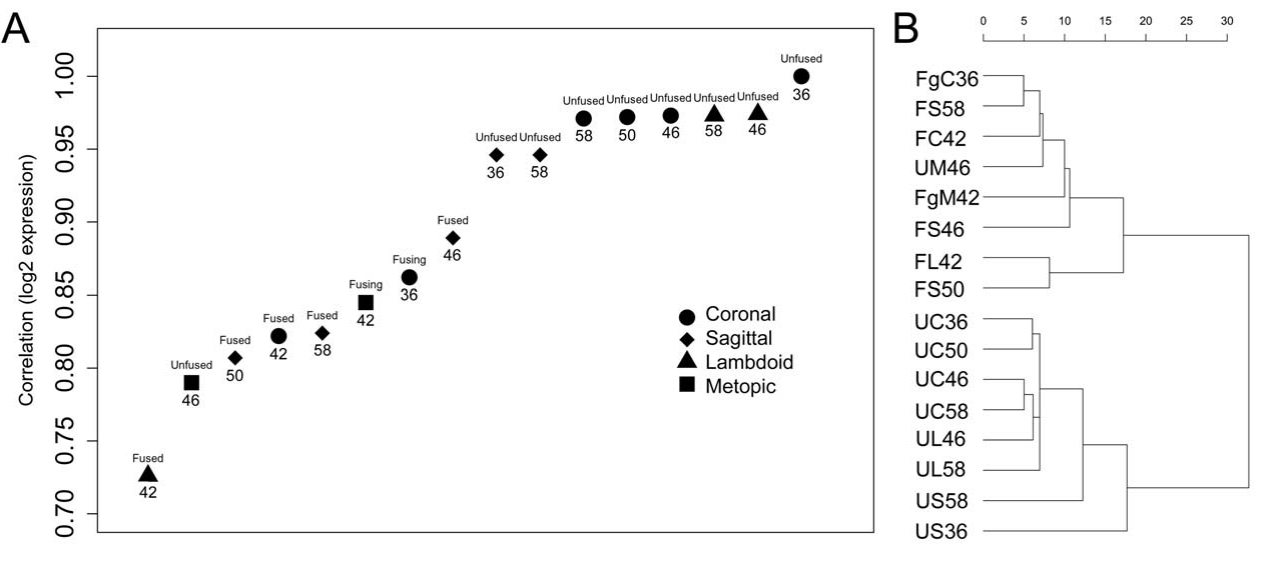
**Microarray sample correlations based on a selected gene list (fused v unfused, *P *< 0.1)**. Correlations are based on genes differentially expressed (P < 0.1) between unfused and fused sutures for combined samples from coronal, lambdoid and sagittal sutures. **A) **Correlation to the unfused coronal suture from patient #36 shows a gradient of correlation between unfused, fusing and fused sutures. The unfused metopic suture groups with fused sutures. Patient number is recorded below data points and state of fusion above data points. **B) **Hierarchical clustering separates suture data into unfused and fusing/fused sutures. Unfused lambdoid and coronal sutures are more related to each other than they are to sagittal sutures. U, unfused; Fg, fusing; F, fused; C, coronal; S, sagittal; L, lambdoid; M, metopic; patient number follows sample identifier.

The neural crest origin of the metopic suture mesenchyme, compared to the predominantly mesodermal origin of the other sutures may result in the metopic suture, exhibiting significantly different expression profiles to the other suture types. We therefore initially analysed differential gene expression between fused and unfused sutures treating metopic sutures separately. Microarray expression data were combined for all unfused (n = 8) and all fusing/fused (n = 6) sutures, from the sagittal, coronal and lambdoid sutures and differential expression was analysed between the two groups. Initially, a subset of differentially expressed probe sets was selected based on those with a multiple testing corrected *P *< 0.1 (n = 84) in order to assess how well the analysis separated the two groups of interest. This minimally-selective P-value was chosen to remove a large number of those genes which were not modulated in the two tissue types. Pair-wise correlation of all samples to an arbitrarily chosen unfused suture sample (#36 non-syndromic, unfused coronal) showed that samples were separated based on stage of fusion using the chosen gene subset, with a gradient of relatedness seen for unfused, fusing, and fused tissues (Fig. 2A). Furthermore, the unfused metopic suture grouped with the fused tissues, suggesting that metopic suture mesenchyme has an expression profile more similar to fused tissue. This result vindicated our exclusion of metopic suture samples from statistical analyses of differential expression between unfused and fused samples. All other unfused sutures showed a very high correlation in expression between themselves, whereas fused and fusing sutures were more disparate in expression profiles. This broader distribution of fusing and fused sutures may indicate that they were undergoing pathologic fusion of different aetiologies and/or that they were at different stages of the fusion process. Additionally, there was evidence for suture-specific expression, with the unfused sagittal sutures being slightly less correlated to unfused coronal sutures than were unfused lambdoid sutures. This difference was later analysed by comparing expression solely between each unfused suture type; however by pooling unfused sutures for the initial analyses we were also able to identify those genes commonly involved in morphogenesis of all sutures.

Hierarchical clustering based on the selected subset of genes clearly showed that suture tissue samples formed two clusters, mirroring two states of fusion: fused/fusing and unfused (Fig. 2B). This suggests that fusing sutures are generally more similar to fused than unfused sutures and that it is appropriate to group them together for analyses. It was also noted that in both plots in Figure 2, sample US36, despite RNA quality concerns, grouped most closely with the other unfused sagittal suture and thus it was appropriate to include this sample in further analyses.

### Genes differentially expressed between fused and unfused sutures

Based on a linear regression analysis of genes differentially expressed between the unfused group of sutures and the group of fusing/fused sutures, 28 genes were significantly (multiple testing corrected *P *< 0.05) differentially expressed (Table 2). Irrespective of P-value, a greater than 2-fold expression difference was found for 829 probe sets; 252 were "increased" and 577 were "decreased" in unfused compared to fusing/fused sutures (Additional file 5). Amongst those genes increased in unfused sutures were *FGFR2*, *TGFB2*, and *epidermal growth factor receptor *(*EGFR*) (Table 3). All have been previously linked with calvarial development and, in the case of *FGFR2*, with craniosynostosis [23-28]. Thirty two of these 829 probe sets (3.9%), representing 24 genes, had a significant difference in expression (multiple testing corrected *P *< 0.05), suggesting that these are important in the morphogenesis of all sutures. All, except one, were increased in unfused sutures. The identification of such a small number of significantly expressed genes across all suture types is likely due to the fact that different suture types, which may have slightly different gene expression profiles, were combined for the analysis. As we did not want to reject any potentially important genes, including those expressed to varying degrees in different sutures, we carried out further analyses using the genes in the 2-fold list, irrespective of their P-value. Importantly, however, we recognise that those genes with a *P *< 0.05 are more likely to be key regulators of suture morphogenesis, rather than specific to one suture type.

**Table 2:** Significantly differentially expressed genes: unfused v fusing/fused sutures.

Gene	GenBank	Name	Fold
*MFAP4*	NM_002404	microfibrillar-associated protein 4	16.50
*IL11RA*	NM_004512	interleukin 11 receptor, alpha	5.90
*RBP4*	NM_006744	plasma retinol binding protein 4	37.38
*AMPH*	NM_001635	amphiphysin	8.27
*INHBA*	NM_002192	inhibin, beta A (activin A, activin AB alpha polypeptide)	6.94
*C1QTNF3*	BC016021	C1q and tumor necrosis factor related protein 3	20.25
*PRELP*	BC032498	proline arginine-rich end leucine-rich repeat protein	10.65
*HDHD1A*	NM_012080	haloacid dehalogenase-like hydrolase domain containing 1A	-1.78
*AGC1*	NM_013227	aggrecan 1 (chondroitin sulfate proteoglycan 1)	6.16
*ANGPTL2*	NM_012098	angiopoietin-like 2	6.67
*C1orf24*	NM_052966	chromosome 1 open reading frame 24	2.04
*FMOD*	NM_002023	fibromodulin	5.41
*OLFM1*	NM_006334	olfactomedin 1	2.55
*FBLN1*	NM_006486	fibulin 1	12.88
*SSPN*	NM_005086	sarcospan	4.40
*CYFIP2*	BC026892	cytoplasmic FMR1 interacting protein 2	-2.68
*MN1*	NM_002430	meningioma 1	3.72
*PTN*	NM_002825	pleiotrophin (osteoblast-specific factor 1)	6.25
*EGFR*	NM_005228	epidermal growth factor receptor	2.71
*TNN*	NM_022093	tenascin N	6.67
*ADCY2*	NM_020546	adenylate cyclase 2 (brain)	2.96
*PDZRN3*	NM_015009	PDZ domain containing RING finger 3	3.71
*SPON1*	BC041974	spondin 1, extracellular matrix protein	4.73
*APP*	NM_000484	amyloid beta (A4) precursor protein	1.83
*AUTS2*	NM_015570	autism susceptibility candidate 2	1.94
*GPC3*	NM_004484	glypican 3	7.13
*HAPLN1*	NM_001884	hyaluronan and proteoglycan link protein 1	7.10
*LHX6*	NM_014368	LIM homeobox 6	-1.80

Twenty-eight genes were identified as being significantly (false discovery rate adjusted *P *< 0.05) differentially expressed in unfused sutures compared to fused/fusing sutures. Genes are listed in order of significance with fold change relative to unfused sutures.

**Table 3:** Selection of genes with fold change between unfused and fused sutures and between each unfused suture site.

Gene Name	Affymetrix ID	Description	Fold U-F	P-value U-F	Unfused Lambdoid	Unfused Coronal	Unfused Sagittal
*Signalling*							
*ANGPTNL2*	213004_at	angiopoietin-like 2	6.67	0.024	1.00	-1.45	-2.87
*EGFR*	201983_s_at	epidermal growth factor receptor	2.71	0.038	1.00	1.02	1.20
*EPHA3*	206071_s_at	EPH receptor A3	2.92	0.480	1.00	-2.06	-5.33
*EPHA4*	206114_at	EPH receptor A4	2.75	0.173	1.00	-1.19	-1.60
*EPHB2*	209589_s_at	EPH receptor B2	2.39	0.093	1.00	1.22	-1.12
*FGFR2*	208228_s_at	fibroblast growth factor receptor 2	3.36	0.294	1.00	-1.42	-3.72
*FZD1*	204451_at	frizzled homolog 1 (Drosophila)	2.13	0.326	1.00	-1.08	-2.41
*IL11RA*	204773_at	interleukin 11 receptor, alpha	5.90	0.003	1.00	1.49	1.32
*TGFB2*	209909_s_at	transforming growth factor, beta 2	3.76	0.096	1.00	-1.01	-2.08
*FGFR1*	222164_at	fibroblast growth factor receptor 1	1.23	0.480	1.00	-1.29	1.12
*FGFR3*	204379_s_at	fibroblast growth factor receptor 3	1.42	0.480	1.00	1.23	1.33
*WNT5A*	205990_s_at	wingless-type MMTV integration site family, member 5A	1.24	0.857	1.00	-1.03	-5.18
*IL17R*	205707_at	interleukin 17 receptor	-2.67	0.480	1.00	-1.25	3.35
*OLFM4*	212768_s_at	olfactomedin 4	-6.69	0.480	1.00	-2.15	6.77
*WIF1*	204712_at	WNT inhibitory factor 1	-3.19	0.480	1.00	-1.27	5.37
*Structural*							
*COL2A1*	213492_at	collagen, type II, alpha 1 (primary osteoarthritis)	7.21	0.390	1.00	-20.66	-3.84
*COL3A1*	215077_at	collagen, type III, alpha 1 (Ehlers-Danlos syndrome type IV)	6.32	0.072	1.00	1.24	-3.84
*COL8A2*	221900_at	collagen, type VIII, alpha 2	11.49	0.092	1.00	-1.23	-12.73
*COL10A1*	217428_s_at	collagen, type X, alpha 1(Schmid metaphyseal chondrodysplasia)	14.65	0.407	1.00	-41.66	-17.28
*FBLN1*	202995_s_at	fibulin 1	12.88	0.025	1.00	-1.39	-1.92
*FMOD*	202709_at	fibromodulin	5.41	0.024	1.00	-1.09	-1.70
*NELL2*	203413_at	NEL-like 2 (chicken)	6.40	0.270	1.00	-4.21	-3.01
*PRELP*	204223_at	proline arginine-rich end leucine-rich repeat protein	10.65	0.009	1.00	1.20	-1.31
*THBS2*	203083_at	thrombospondin 2	6.62	0.069	1.00	-1.26	-2.47
*COL1A2*	202404_s_at	collagen, type I, alpha 2	1.05	0.480	1.00	-1.01	-1.06
*CORO1A*	209083_at	coronin, actin binding protein, 1A	-5.08	0.480	1.00	-1.05	11.73
*Transcription*							
*BHLH3*	221530_s_at	basic helix-loop-helix domain containing, class B, 3	5.95	0.093	1.00	-2.23	-4.76
*CART1*	206837_at	cartilage paired-class homeoprotein 1	2.06	0.533	1.00	16.21	2.38
*HLF*	204753_s_at	hepatic leukemia factor	3.54	0.210	1.00	-2.55	-6.81
*JUN*	201465_s_at	v-jun sarcoma virus 17 oncogene homolog (avian)	4.41	0.161	1.00	3.14	2.50
*PITX2*	207558_s_at	paired-like homeodomain transcription factor 2	3.45	0.279	1.00	1.63	-2.08
*SIX2*	206510_at	sine oculis homeobox homolog 2 (Drosophila)	2.72	0.136	1.00	-1.09	-1.70
*FOS*	209189_at	v-fos FBJ murine osteosarcoma viral oncogene homolog	1.40	0.745	1.00	14.96	4.13
*FOSB*	202768_at	FBJ murine osteosarcoma viral oncogene homolog B	1.83	0.695	1.00	54.37	20.22
*FOXD1*	206307_s_at	forkhead box D1	1.49	0.499	1.00	-4.71	-5.17
*JUNB*	201473_at	jun B proto-oncogene	1.28	0.703	1.00	3.61	2.95
*MEOX2*	206201_s_at	mesenchyme homeo box 2 (growth arrest-specific homeo box)	1.66	0.495	1.00	-3.99	-7.74
*MSX2*	210319_x_at	msh homeo box homolog 2 (Drosophila)	1.95	0.464	1.00	-1.14	-2.23
*TWIST1*	213943_at	twist homolog 1 (Saethre-Chotzen syndrome)	1.50	0.476	1.00	-1.20	-1.27
*LHX6*	219884_at	LIM homeobox 6	-1.80	0.050	1.00	-1.07	-1.03
*SHOX2*	210135_s_at	short stature homeobox 2	-3.44	0.480	1.00	-1.00	4.50
*PAX5*	221969_at	paired box gene 5 (B-cell lineage specific activator)	-4.20	0.480	1.00	-1.69	6.25
*Catalytic Activity*							
*ROR1*	205805_s_at	receptor tyrosine kinase-like orphan receptor 1	2.26	0.095	1.00	1.21	-1.14
*HDHD1A*	203974_at	haloacid dehalogenase-like hydrolase domain containing 1A	-1.78	0.018	1.00	1.05	1.05
*ALOX5*	204446_s_at	arachidonate 5-lipoxygenase	-5.55	0.480	1.00	-1.30	6.76
*ANXA3*	209369_at	annexin A3	-7.17	0.480	1.00	-1.30	6.76
*Transporter*							
*C1QTNF3*	220988_s_at	C1q and tumor necrosis factor related protein 3	20.25	0.007	1.00	-1.52	-4.92
*RBP4*	219140_s_at	retinol binding protein 4 (plasma) KIAA1922	37.38	0.003	1.00	-1.35	-2.53
*APOC1*	204416_x_at	apolipoprotein C-I	-2.01	0.480	1.00	1.08	5.06
*Binding*							
*AGC1*	217161_x_at	aggrecan 1 (chondroitin sulfate proteoglycan 1)	6.16	0.024	1.00	-1.25	-1.56
*CCND1*	208711_s_at	cyclin D1	2.13	0.449	1.00	1.38	2.24
*GPC3*	209220_at	glypican 3	7.13	0.050	1.00	1.26	-3.34
*HAPLN1*	205523_at	hyaluronan and proteoglycan link protein 1	7.10	0.050	1.00	-1.96	-4.01
*INHBA*	210511_s_at	inhibin, beta A (activin A, activin AB alpha polypeptide)	6.94	0.006	1.00	-1.36	-3.03
*MFAP4*	212713_at	microfibrillar-associated protein 4	16.50	0.001	1.00	1.32	-1.86
*OGN*	218730_s_at	osteoglycin (osteoinductive factor, mimecan)	5.71	0.166	1.00	-1.41	-4.65
*POSTN*	214981_at	periostin (osteoblast specific factor 2)	5.87	0.101	1.00	-1.16	-1.60
*PTN*	209466_x_at	pleiotrophin (osteoblast specific factor 1)	6.25	0.031	1.00	-1.14	-1.78
*S100A10*	200872_at	S100 calcium binding protein A10 (annexin II ligand)	2.01	0.096	1.00	-1.04	-1.60
*CCND3*	201700_at	cyclin D3	-2.60	0.480	1.00	-1.00	3.62
*FCN1*	205237_at	ficolin (collagen/fibrinogen domain containing) 1	-5.45	0.480	1.00	-1.46	12.39
*FGR*	208438_s_at	Gardner-Rasheed feline sarcoma viral (v-fgr) oncogene homolog	-3.03	0.480	1.00	-1.13	5.75
*S100A12*	205863_at	S100 calcium binding protein A12 (calgranulin C)	-8.86	0.480	1.00	-2.63	15.19
*Enzyme regulator*							
*MMP14*	202827_s_at	matrix metalloproteinase 14 (membrane-inserted)	2.39	0.226	1.00	-1.22	-1.84
*TIMP3*	201149_s_at	tissue inhibitor of metalloproteinase 3	2.22	0.467	1.00	-1.71	-3.43
*CASP1*	211366_x_at	caspase 1, apoptosis-related cysteine protease	-2.73	0.165	1.00	-1.30	1.01
*MME*	203434_s_at	membrane metallo-endopeptidase (CD10)	-3.23	0.476	1.00	-2.35	-1.29
*MMP8*	207329_at	matrix metalloproteinase 8 (neutrophil collagenase)	-5.81	0.480	1.00	-2.36	1.69
*RASGRP2*	208206_s_at	RAS guanyl releasing protein 2 (calcium and DAG-regulated)	-2.60	0.480	1.00	1.02	3.02
*Unknown Function*							
*CYFIP2*	220999_s_at	cytoplasmic FMR1 interacting protein 2	-2.68	0.031	1.00	1.14	1.09

Gene expression fold change (U-F) is presented for unfused sutures (n = 8) compared to fused/fusing sutures (n = 6), with corresponding P-values, and for combined unfused coronal (n = 4) and unfused sagittal (n = 2) sutures compared to unfused lambdoid sutures (n = 2).

To further categorise the 2-fold differentially-expressed gene list, gene ontology (GO) over-representation was analysed. Biological processes enriched in the 2-fold gene list are shown in Table 4. Genes with higher expression in unfused sutures were found to significantly over-represent processes such as mesoderm formation, skeletal development, cell adhesion, cell surface receptor signalling, and extracellular matrix organisation, consistent with genes involved in regulating suture morphogenesis. Surprisingly, we also noted an extremely significant over-representation within those genes with higher expression in fusing/fused sutures of genes involved in the response to biotic stimuli (*P *= 5.73 × 10^-41^) and the immune response (*P *= 1.61 × 10^-34^). As fold change is not the only useful characteristic, GO over-representation analysis was also conducted irrespective of fold change for all probe sets with a minimally selective *P *< 0.25 (n = 261) and similar categories were identified.

**Table 4:** Gene Ontology analysis: unfused compared to fused sutures

Biological process increased in sutures:	Unfused	Fused	P-value
Cell adhesion	32		1.95 × 10^-12^
Cell matrix adhesion	4		6.53 × 10^-3^
Cell communication			
Cell surface receptor linked signal transduction	25		4.45 × 10^-3^
Dopamine metabolism	2		6.63 × 10^-4^
Phosphoinsitide-mediated signalling		8	4.58 × 10^-3^
Cell differentiation			
Heme biosynthesis		3	6.96 × 10^-3^
Lymphocyte differentiation		6	1.27 × 10^-3^
Cellular physiological process			
Anion transport	11		4.92 × 10^-6^
Cell cycle		42	4.72 × 10^-5^
Cell motility	11		2.70 × 10^-4^
Extracellular matrix organisation and biosynthesis	4		1.11 × 10^-3^
Microtubule based process		10	7.35 × 10^-3^
Regulation of phosphorylation	3		5.30 × 10^-3^
Morphogenesis	18		5.34 × 10^-4^
Cellular morphogenesis	9		5.60 × 10^-3^
Mesoderm formation	2		4.46 × 10^-3^
Organ morphogenesis	8		7.46 × 10^-3^
Organ development	23		3.45 × 10^-7^
Cartilage condensation	2		5.89 × 10^-3^
Eye development	3		4.98 × 10^-4^
Muscle development	6		2.52 × 10^-3^
Skeletal development	11		1.18 × 10^-6^
Regulation of development			
Negative regulation of development	3		2.28 × 10^-3^
Response to abiotic stimulus			
Response to chemical stimulus		20	7.31 × 10^-3^
Response to reactive oxygen species		3	5.21 × 10^-3^
Response to biotic stimulus		116	5.73 × 10^-41^
Defence response to bacteria		11	5.2 x10^-9^
Defence response to fungi		3	1.65 × 10^-3^
Response to virus		7	9.11 × 10^-3^
Immune response		100	1.61 × 10^-34^
Humoral immune response		22	1.57 × 10^-8^
Inflammatory response		23	3.08 × 10^-7^

Classification of gene ontologies (GO) significantly over-represented (*P *< 0.01) for genes at least 2 fold differentially expressed between unfused and fused sutures, listed under level 3 GO category headings. Not all level three GO categories listed were significantly over-represented and thus do not have a P-value

Gene Set Enrichment Analysis (GSEA) was then used to asses the significance of this set of differentially expressed genes at the molecular level. The ranked list of 2-fold differentially expressed genes was compared with a curated database consisting of molecular pathways and publicly available microarray experiments (Additional file 6). Such a comparison identifies which molecular pathways share a group of genes with our identified gene list, providing a potential insight into involved biological networks. Those gene sets which were significantly correlated (multiple testing corrected *P *< 0.05) to genes increased in unfused sutures included genes with activating transcription factor 3 (ATF3) and lymphoid enhancer-binding factor 1 (LEF1) binding motifs within 2 kb of their transcription start sites, genes up-regulated by TGFβ, genes up-regulated in haematopoietic stem cells, genes up-regulated in CD31 negative stromal stem cells which differentiate into bone cells, and genes down-regulated upon Cytomegalovirus (CMV) infection. This final gene set makes a connection between those genes down-regulated during suture fusion (i.e. up-regulated in unfused sutures) and genes down-regulated during infection. This integrated well with the gene sets which were significantly correlated with those genes increased during fusion; these included genes up-regulated in liver in graft verses host disease (GVHD; particularly genes associated with attraction and activation of donor T-cells), genes up-regulated in pulpal tissue from carious teeth, and genes up-regulated during retinoic acid induced promyelocytic differentiation. These observations are consistent with our previous GSEA observations in a microarray comparison between fused sutures tissue and de-differentiated explant cells, where we again found an increase in expression of immune response genes in fused suture tissues [29]. It is possible that these immune response genes reflect the formation of bone marrow within the fused bone matrix. In support of this, microscopy revealed a large accumulation of lymphocytes and other white blood cells within the calvarial bones (Additional file 7). A second explanation may be that premature fusion is functionally associated with an immune response to infection, either directly or indirectly, as it is known that various immunoregulatory cytokines influence bone homeostasis and that osteoblasts may facilitate immune responses by producing immunomodulatory molecules (reviewed in [30,31]).

Results were then analysed on a gene-based level. One of the families of genes which were significantly over-represented in unfused sutures was Eph/ephrin signalling molecules. These form a pathway recently invoked in causing craniosynostosis [32,33]. Specifically, we found that three ephrin receptor genes had higher expression in unfused sutures (*EPHA3*, 2.9-fold; *EPHA4*, 2.8-fold; *EPHB2*, 2.4-fold). Multiple genes from several other gene families were also increased in unfused sutures (Table 3). These include small leucine-rich proteoglycans (SLRPs), a group of secreted proteins that are known to be involved in cartilage and bone formation through facilitating collagen fibril binding to the EMC [34,35] and regulating TGFβ activity by sequestering TGFβ in the ECM, thus preventing binding to cell surface receptors [36]. Such genes were, *proline arginine-rich end leucine-rich repeat protein *(*PRELP*, 10.7-fold), *osteoglycin *(*OGN*, 5.7-fold), *fibromodulin *(*FMOD*, 5.4-fold) and *decorin *(*DCN*, 2.0-fold).

A large over-representation of collagen genes was also observed. In particular, *collagen type II, III*, *VI*, *VIII*, *X*, and *XI *were all up-regulated in unfused sutures. Interestingly, Collagen type II and X are generally associated with cartilage formation, and would not be expected to be expressed during intramembranous ossification. However, a number of other cartilage-specific genes were also increased in unfused sutures (*AGC1*,6.2-fold; *HAPLN1*, 7.1-fold; *CART1*, 2.1-fold) suggesting that there may be a role for cartilage in calvarial suture morphogenesis. Additional secreted matrix proteins that were over-expressed in unfused suture tissue included *pleiotrophin *(*PTN*, also known as *osteoblast specific factor 1 *(*OSF1*)) and *periostin *(*POSTN*, *osteoblast specific factor 2 *(*OSF2*)). *PTN *has been identified in osteoblasts undergoing early stages of differentiation and is a potent regulator of osteoblast proliferation, recruitment, and differentiation [37]. *POSTN *is also expressed by early osteoblasts and is a target of Twist1 transcriptional regulation in mice [38]. A number of proteases and protease inhibitors were also differentially expressed between unfused and fused suture tissue including, *MMP2 *(2.0-fold), *MMP14 *(2.4-fold), *MMP8 *(5.8-fold), *MME *(-3.23-fold), *SERPINA1 *(-2.7-fold), *SERPINB1 *(-3.6-fold) and *TIMP3 *(2.3-fold) (Additional file 5).

Numerous genes involved in Wnt signalling were also identified; *glypican 3 *(*GPC3*, 7.1-fold) and *frizzled 1 *(*FZD1*, 2.1-fold) were increased in unfused sutures, while *WNT inhibitory factor 1 *(*WIF1*, 3.2-fold) was increased in fused sutures. Previously, we have identified an up-regulation of *WIF1 *in human fused suture tissue when comparing *in vivo *expression to expression of de-differentiated explant cells [29]. These results are consistent with the recent observations that activation of canonical Wnt signalling is important in osteoblast expansion and differentiation [39], and that antagonist of Wnt signalling are essential to initiate terminal osteoblasts differentiation [40].

A number of the genes differentially expressed between fused, fusing, and unfused sutures which had a large significant (*P *< 0.05) fold change (Table 2) had not been previously identified to be expressed in human calvaria. Of particular interest were *retinol-binding protein 4 *(*RBP4*, 37.4-fold, *P *= 0.003), *C1q and tumour necrosis factor related protein 3 *(*C1QTNF3*, 20.3-fold, *P *= 0.007), *microfibrillar-associated protein 4 *(*MFAP4*, 16.5-fold, *P *= 0.001), *PRELP *(10.7-fold, *P *= 0.009), *GPC3 *(7.1-fold, *P *= 0.05), *tenascin N *(*TNN*, 6.7-fold, *P *= 0.038), *pleiotrophin *(*PTN*, 6.3-fold, *P *= 0.031) and *interleukin 11 receptor alpha *(*IL11RA*, 5.9-fold, *P *= 0.003). The significant expression of all these genes for the combined suture comparison suggests that they are likely to be key regulators of morphogenesis in all sutures.

### Unfused sagittal sutures have a lower expression of the 'unfused' class of genes

**Figure 3:**
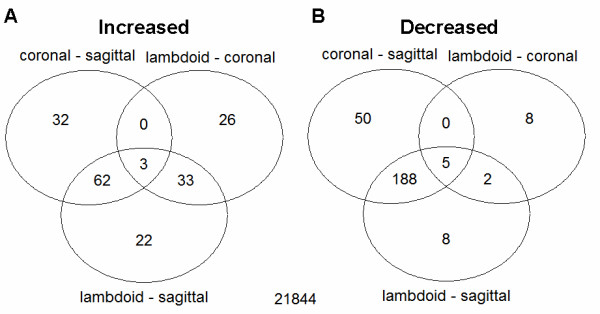
**Venn diagram of 3-way unfused suture comparison results**. Global expression differences were compared for 3 pair-wise comparisons (coronal vs sagittal, lambdoid vs coronal, lambdoid vs sagittal). The sagittal suture showed the greatest difference in gene expression to the other two sutures, followed by the lambdoid suture. **A) **Increased genes. 62 genes had similar increased expression in coronal and lambdoid sutures compared to sagittal sutures and 33 genes had increased expression in lambdoid sutures and similar expression in coronal and sagittal sutures. **B) **Decreased genes. 188 genes had similar decreased expression in unfused coronal and lambdoid sutures compared to unfused sagittal sutures and 2 genes had decreased expression in lambdoid sutures and similar expression in coronal and sagittal sutures.

To analyse the effect of suture type on gene expression we used two methods of analysis to compare global expression data between unfused coronal, sagittal, and lambdoid sutures. We then compared results from the two methods to identify the most robust set of differentially expressed genes. For the 3-way analysis we used a linear modelling approach to jointly perform three pair-wise comparisons: coronal vs sagittal, lambdoid vs sagittal, and lambdoid vs coronal and we then identified where these groups overlapped (Fig. 3). This pooled approach provides greater sensitivity and statistical power over multiple direct comparisons. Unfused sagittal sutures were found to have 340 probe sets differentially expressed when compared to coronal sutures and 323 when compared to lambdoid sutures, while the coronal and lambdoid sutures only had 77 probes sets differentially expressed between each other. Furthermore, 250 of those probe sets differentially expressed in unfused sagittal were not differentially expressed between the coronal and lambdoid sutures. This supported the higher correlation of gene expression of differentially expressed genes between unfused coronal and lambdoid sutures seen in Figure 2. Of these 250 probe sets, 62 were increased in unfused coronal and lambdoid sutures and 188 were decreased, compared to the sagittal sutures (Fig. 3). The 3-way analysis also showed that 35 probe sets were uniquely expressed in unfused lambdoid sutures compared to coronal and sagittal sutures (33 increased and 2 decreased).

To control for patient-specific effects we performed a set of separate pair-wise comparisons using a matched pairs design. In this case we first restricted analysis to patients with a sample from each of the sutures of interest and then performed analysis on the within patient differences. A list of significant genes with *P *< 0.01 was produced from each pair-wise comparison (Additional files 8, 9, 10).

**Figure 4:**
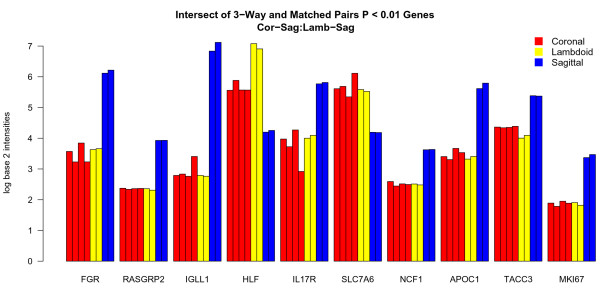
**Top ten genes differentially expressed between unfused coronal, lambdoid and sagittal sutures**. Genes commonly identified to be differentially expressed (*P *< 0.01) in unfused coronal and lambdoid sutures compared to unfused sagittal sutures, using two methods of microarray data analysis: 3-way and matched-pairs. Eight genes (*FGR*, *RASGRP2*, *IGLL1*, *IL17R*, *NCF1*, *APOC1*, *TACC3*, and *MKI67*) have higher expression in unfused sagittal sutures and two (*HLF *and *SLC7A6*) have lower expression compared to unfused coronal and lambdoid sutures. *HLF *shows a gradient of expression, highest in lambdoid, then coronal, then sagittal sutures, whereas the other genes show similar expression in coronal and lambdoid sutures. For each gene listed, sutures are in patient order: coronal (#36, #46, #50, #58), lambdoid (#46, #58), sagittal (#36, #58).

The combination of the 3-way and pair-wise comparisons identified 100 probe sets significantly (*P *< 0.01) differentially expressed in unfused sagittal sutures compared to unfused coronal and lambdoid sutures. Amongst the top ten genes, seven had higher expression in unfused sagittal sutures and two had lower expression compared to coronal and lambdoid sutures (Fig. 4). Outside the top ten, genes with significantly decreased expression in unfused sagittal sutures were *FGFR2*, *MSX2*, *GPC3*, and *WNT5A*, while the Wnt inhibitor *WIF1 *had increased expression. The observed trend was that unfused sagittal sutures have a lower expression of those genes typically associated with an unfused suture state and a higher expression of genes associated with suture fusion (Table 3).

### Unfused coronal and sagittal sutures have differential expression of transcription factors compared to lambdoid sutures

Analysis of the unfused-suture comparison data with respect to genes differentially expressed by unfused lambdoid sutures identified a large number of transcription factors. Those down-regulated with respect to coronal and sagittal sutures included *FOS*, *FOSB*, *JUN*, *JUNB*, and *CART1*; all having a tendency for greater expression in the coronal suture (Table 3). Those genes with higher expression in unfused lambdoid sutures included transcription factors *FOXD1*, *MEOX2*, *HLF *and *BHLH3*.

### Microarray results validated by real-time quantitative RT-PCR and Western blot

Results from the microarray analysis were validated by qRT-PCR for 11 of the most highly expressed and significantly differentially expressed genes: eight genes increased in unfused suture tissue, *RBP4*, *C1QTNF3*, *PRELP*, *GPC3*, *PTN*, *FMOD*, *COL3A1*, and *COL8A2*; and 3 genes increased in fusing/fused suture tissue, *WIF1*, *ANXA3*, and *CYFIP2*. The Affymetrix probe set for *C1QTNF3 *targeted two transcripts and therefore two transcript-specific primers sets were designed. Gene expression was analysed using 10 of the same RNA samples which underwent microarray analysis (Table 1). A linear correlation was calculated for each transcript for the comparison of expression values obtained by qRT-PCR and microarray analysis (Table 5). An average correlation of 90% was observed for all genes analysed, validating the microarray results. Three primer sets had a correlation coefficient smaller than 75%; however, two of these primer sets did not amplify all isoforms detected by their corresponding Affymetrix probe set. The third primer set was designed to detect the long isoform of *C1QTNF3*. While this had a correlation of 74%, the short isoform *C1QTNF3 *primers had 99% correlation, suggesting that it is the short isoform of *C1QTNF3 *that is differentially expressed.

**Table 5:** Linear correlation between microarray and qRT-PCR data.

Gene	Probe Set	Slope	Correlation
*ANXA3*	209369_at	0.881	0.978
*WIF1*	204712_at	0.850	0.982
*CYFIP2*	220999_s_at	0.785	0.748
*PTN*	211737_x_at	1.063	0.905
*PRELP*	204223_at	0.755	0.938
*FMOD*	202709_at	0.867	0.851
*C1QTNF3 *long isoform	220988_s_at	1.774	0.738
*C1QTNF3 *short isoform	220988_s_at	0.806	0.990
*RBP4*	219140_s_at	1.100	0.968
*GCP3*	209220_at	1.036	0.929
*COL8A2*	221900_at	1.261	0.854
*COL3A1*	215077_at	0.767	0.709
Average Correlation			0.896

The average correlation for all probe sets is approximately 90%, not including the correlation of the long isoform of C1QTNF3. The short isoform of C1QTNF3 had 99% correlation, indicating that the short and not the long isoform of C1QTNF3 is differentially expressed between unfused and fused sutures.

**Figure 5:**
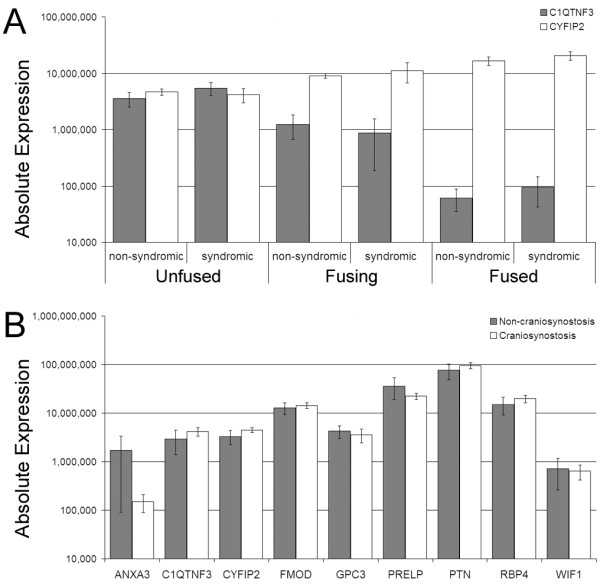
**qRT-PCR expression data comparing syndromic, non-syndromic and non-craniosynostosis samples**. **A) **No significant difference in expression exists between suture samples from syndromic and non-syndromic craniosynostosis patients. *C1QTNF3 *is decreased during fusion and *CYFIP2 *is increased during fusion, irrespective of aetiology. Samples from coronal, sagittal, lambdoid, and metopic sutures were combined for analysis. Samples numbers (left to right) are n = 8, 4, 5, 4, 7, 7. **B) **Unfused sutures from craniosynostosis patients (n = 12) and individuals without craniosynostosis (n = 6) show no significant difference (*P *< 0.05) in expression for all genes analysed. Absolute expression values represent molecules per ng cDNA. Mean expression ± SEM is shown.

To further examine the differential expression of genes between fused, fusing and unfused sutures identified by microarray analysis we used qRT-PCR to quantify expression of all genes noted above using the 10 validation samples and 25 additional samples from 13 new patients (Table 1). Although the initial microarray hierarchical clustering analyses (Fig. 2, Additional file 4) indicated there was limited difference in whole genome expression between non-syndromic and syndromic samples, this analysis only included samples from one syndromic patient. We therefore extended this comparison by analysing qRT-PCR data obtained from the larger cohort of patients which included 7 syndromic patients, and 11 non-syndromic patients in total. No significant difference (*P *< 0.05) in gene expression was seen between non-syndromic and syndromic samples, when separated into unfused, fusing, and fused states (Fig. 5A, Additional file 11). This result clearly demonstrates that samples of different aetiologies can be combined to investigate the general mechanisms of craniosynostosis.

As the unfused suture samples which underwent microarray and qRT-PCR analyses were obtained from patients with craniosynostosis, there is the possibility that their gene expression profiles do not truly represent an unfused suture from an individual without craniosynostosis. Consequently, the expression of the above mentioned genes were analysed in unfused coronal, lambdoid, and metopic sutures obtained from similar age-matched individuals who were undergoing transcranial surgery for reasons other than craniosynostosis (Table 1). No significant difference (*P *< 0.05) in gene expression was observed for these unfused non-craniosynostosis sutures, compared to unfused sutures from individuals with craniosynostosis (Fig. 5B). This provides proof of principle that the analysis of gene expression profiles from unfused sutures from craniosynostosis patients is useful in the study of suture morphogenesis.

**Figure 6:**
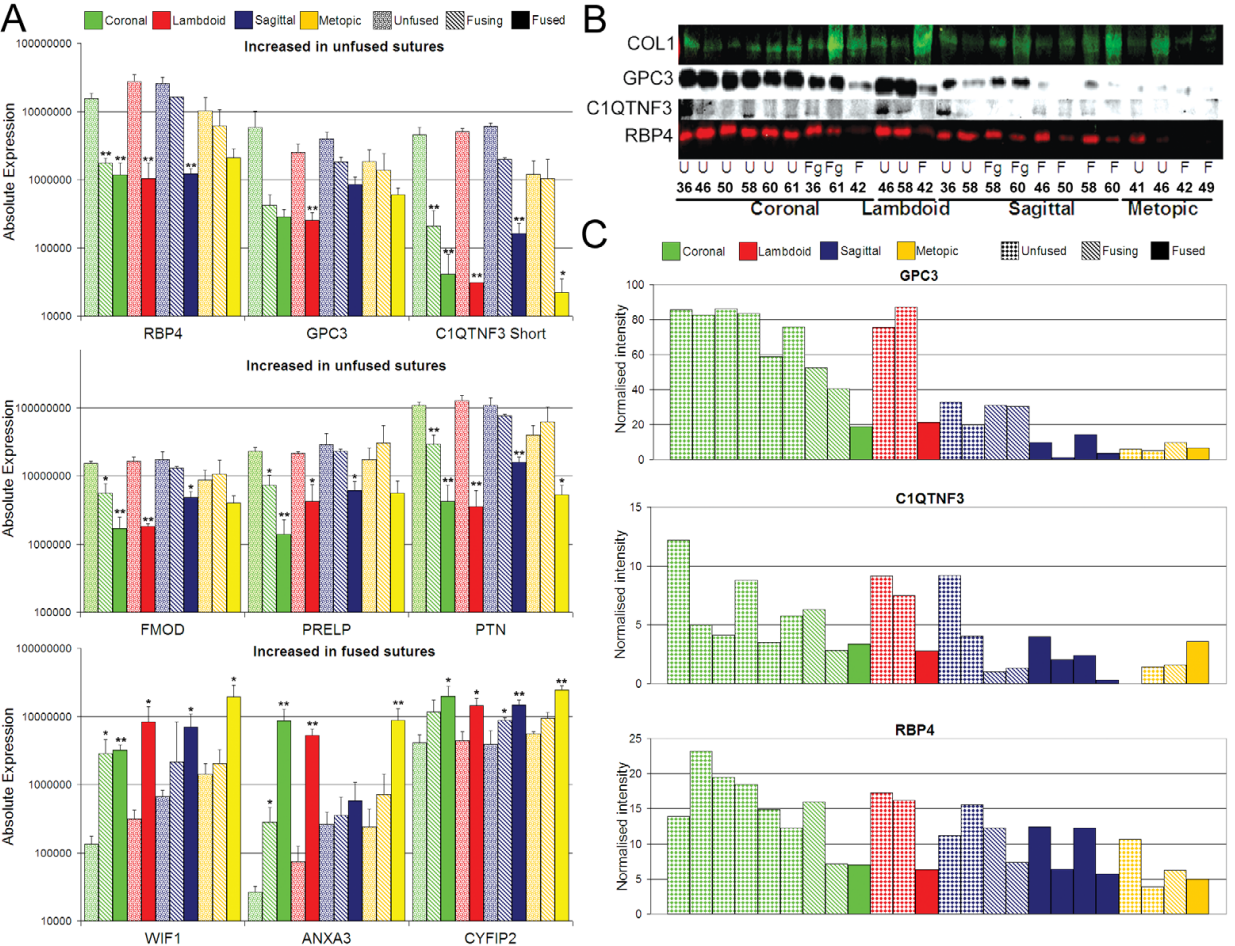
**mRNA and protein validation of differential expression identified by microarray analysis**. **A) **Real-time qRT-PCR analysis of six genes with increased expression in unfused sutures (*RBP4*, *GPC3*, *C1QTNF3 short isoform*, *FMOD*, *PRELP*, and *PTN*) and three genes with increased expression in fused sutures (*WIF1*, *ANXA3*, and *CYFIP2*) for unfused, fusing and fused suture tissue isolated from sagittal, coronal, lambdoid and metopic sutures. Significant differential expression (*P *< 0.05, *; *P *< 0.01, **) was analysed for fusing and fused sutures compared to unfused sutures. Mean expression + SEM is shown; n = 3 for all comparisons, except fused sagittal (n = 5), fused metopic (n = 4), and fused lambdoid (n = 2). Absolute expression values represent molecules per ng cDNA. **B) **Western blot analysis of individual protein samples in the order seen in (C), for collagen type 1 (COL1), GPC3, C1QTNF3 and RBP4. **C) **Densitometry analysis of western blots normalised to COL1 expression.

**Figure 7:**
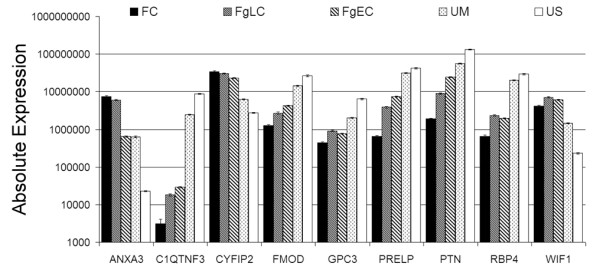
**Within patient comparison of gene expression in fused, fusing, and unfused sutures**. Gene expression was analysed in five suture samples from an Apert syndrome patient (#90) by qRT-PCR. A gradient of expression is seen for all genes from unfused sagittal (US), to unfused metopic (UM), early fusing coronal (FgEC), late fusing coronal (FgLC), and fused coronal (FC) sutures. *ANXA3 *and *CYFIP2 *are increased highest in fused sutures, *WIF1 *in fusing sutures and all other genes have highest expression in unfused sagittal sutures. Unfused Metopic sutures show higher expression of genes increased during fusion and a lower expression of genes increased in other unfused sutures. Absolute expression values represent molecules per ng cDNA. Error bars represent SEM for triplicate technical replicates.

qRT-PCR data was then compared between unfused, fusing, and fused samples from each suture site, combing non-syndromic and syndromic patients together. Differential expression profiles were observed for all 11 genes analysed, although the level of differential expression varied between suture sites (Fig. 6A, Additional file 12). The greatest difference in expression was observed for coronal sutures, followed closely by lambdoid sutures, while metopic sutures had, in general, the smallest changes in expression between unfused and fused sutures. This later observation is likely due to the finding that unfused metopic sutures generally had a lower level of expression of genes increased in unfused sutures (eg. *C1QTNF3*, *FMOD *and *PTN*) and a higher expression of genes which were increased in fused sutures (eg. *WIF1 *and *ANXA3*). Unfused sagittal sutures also showed higher expression than unfused coronal and lambdoid sutures for those genes increased in fused sutures (*ANXA3 *and *WIF1*) (Fig. 6A). These combined patient results confirmed the suture-specific analyses outlined earlier (Table 3, Fig. 2). The qRT-PCR validation experiment also demonstrated a variable gradient of expression between unfused, fusing, and fused samples for all genes analysed (Fig. 5A and Fig. 6A). However, this gradient of expression is best analysed using samples isolated from the same sutures from the same patient that are undergoing various stages of fusion. Figure 7 shows the qRT-PCR data from 5 samples isolated from one Apert syndrome patient (#90). Two fusing coronal samples were isolated from this patient, one during the early stages and one during the later stages of fusion, along with a fully fused coronal suture and unfused sagittal and metopic sutures. The data shows that *ANXA3 *and *CYFIP2 *are increased in fusing sutures, but are further increased in fully fused sutures, whereas *WIF1 *has the greatest expression in fusing sutures and is slightly decreased once the suture is fully fused, however this level remains above unfused sutures. This subtle difference cannot be seen in Figure 6A where fusing sutures of different stages from different patients are grouped together. Figure 7 also again highlights the difference in expression of unfused metopic sutures, being closer in expression to fusing sutures than other unfused sutures for a number of genes.

Differential protein expression was assessed by Western blot analysis for three genes with increased expression in unfused sutures, *RBP4*, *C1QTNF3 *and *GPC3 *(Fig. 6B and 6C). Microarray results indicated that *collagen type I alpha 2 *was the most abundant transcript and was not differentially expressed between unfused and fused suture tissues (1.05 fold, Table 3). Protein expression was therefore normalised to COL1 for comparative quantification. All three proteins were differentially expressed in a similar pattern to that observed for the RNA expression data, with a decreasing gradient of expression observed for unfused, fusing, and then fused samples. Again, lower expression of each protein was observed in unfused metopic sutures. Higher protein expression was observed in two fused sagittal sutures (#46 and #58) compared to two other fused sagittal sutures (#50 and #60). This may be explained in part by tissue structure. During sample preparation it was noted that #46 and #58 sutures were very thin, flat bones more representative of developed calvaria, while #50 and #60 were more archetypal, having enlarged fused-suture ridges [5].

### Unfused Lambdoid sutures express cartilage-specific markers

**Figure 8:**
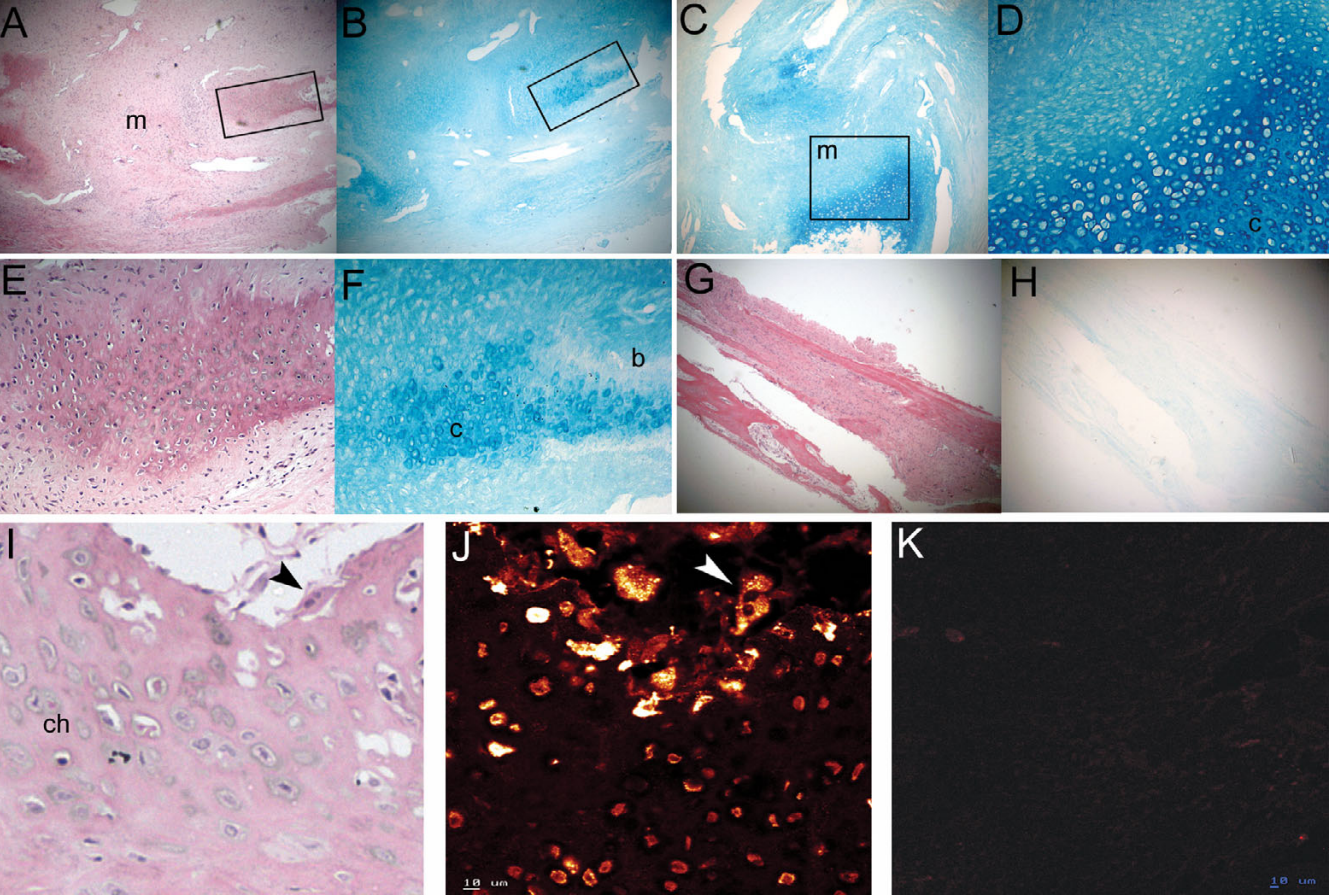
**Cartilage localisation in unfused sutures**. **A-B) **Serial H&E and Alcian blue stain of right unfused lambdoid suture (#83) showing cartilage (boxed regions expanded in panels E and F, respectively) on either side of suture mesenchyme (m). **C) **Alcian blue stain of left unfused lambdoid suture (#83) showing cartilage fronts (box) surrounding suture mesenchyme. **D) **Cartilage front from panel C (box) showing proliferating (stacked cells) and hypertrophic (cells with enlarged lacunae) chondrocytes in a cartilage (c) matrix. **E-F) **Enlarged views of boxed regions in A and B, respectively showing H&E (E) and Alcian blue (F) stain of right unfused lambdoid suture (#83) and highlighting cartilage interspersed with calcified bone (b, dark pink). Hematoxylin stains calcified matrix darker. **G-H) **Serial H&E (G) and Alcian blue (H) staining of an unfused coronal suture (#83) showing no staining of cartilage in (H). **I-J) **H&E (I) and confocal immunofluorescence for Collagen type X (J) detected weak localisation (orange) in hypertrophic chondrocytes (ch), with intense (yellow) punctate localisation in osteoclasts (multi-nucleated cells, arrowhead) adjacent to the cartilage matrix of unfused lambdoid sutures (#83). **K) **Collagen type X protein was not detected in osteogenic fronts of unfused coronal sutures (#83). Magnification: A-C, G-H: X3.2; D-F: X12.5; Scale: I-K: 10 μm.

Increased expression of *COL10A1 *and *COL2A1 *was identified in unfused lambdoid sutures and one (#58) of the two unfused sagittal sutures compared to all other samples (Table 3). The unfused sagittal suture from patient #58 was taken from the extreme posterior portion of the suture very close to the lambdoid suture (Fig. 1A). In all other gene expression analyses this sample grouped with the other unfused sagittal suture, verifying its correct classification as sagittal suture tissue. The expression of cartilage-specific collagens suggested, contrary to conventional thinking, that cartilage may play a role in human suture morphogenesis. Histological analysis of fused and unfused lambdoid, coronal, and sagittal sutures from additional patients to those used for microarray analyses (Table 1), identified cartilage only in unfused lambdoid sutures (Fig. 8). Cartilage was found at the tips of the osteogenic fronts which protruded into the suture mesenchyme. This cartilaginous region was composed of what histologically appeared to be a region of proliferating and hypertrophic chondrocytes, and was seen to protrude into the osteoblastic region where calcification was occurring to form calvarial bone (Fig. 8A–8F). The spatial expression of COLX protein, which is a specific marker of hypertrophic chondrocytes [41], was investigated by confocal immunofluorescence (Table 1). COLX was found to be highly abundant in osteoclasts, which were localised to the edges of the cartilage matrix adjacent to the bone matrix. COLX was also expressed, but more weakly, by chondrocytes in the cartilage matrix (Fig. 8I, J). COLX expression was not identified in any other suture type (Fig. 8K).

### Different spatial localisation of RBP4 and GPC3 in unfused sutures

**Figure 9:**
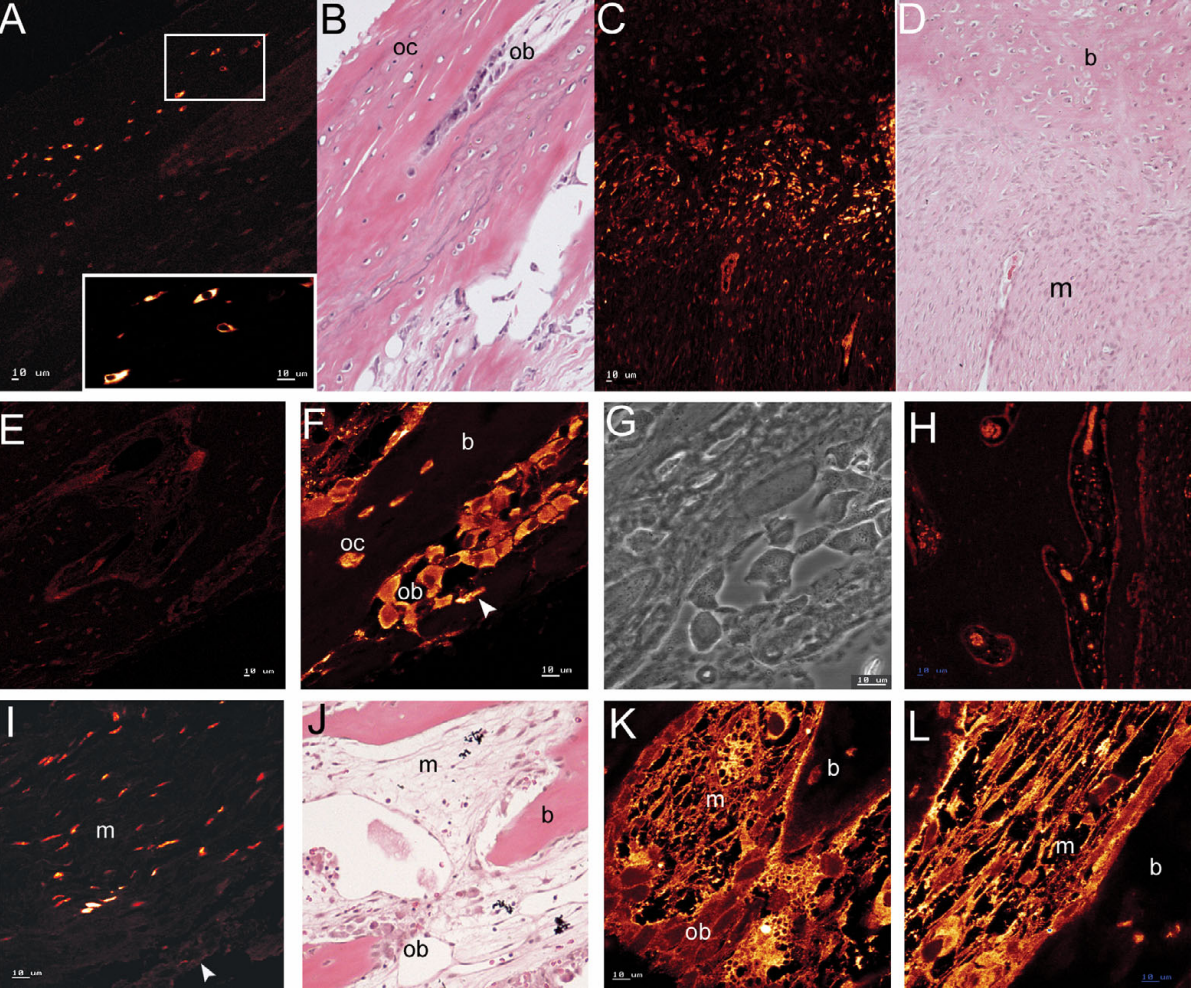
**Localisation of RBP4 and GPC3 in suture tissue**. **A-B) **Immunofluorescence and H&E stain showing intense localisation (yellow) of RBP4 in the cytoplasm of osteocytes (oc) in ectocranial surface bone (unfused coronal suture, #83). **C-D) **Serial immunofluorescence (C) and H&E sections (D) showing RBP4 located in cells in the region between calcified tissue (b) and mesenchyme (m) (unfused left lambdoid suture, #83). **E) **RBP4 was not detected on the endocranial surface of unfused sutures (coronal, #83). **F) **RBP4 was localised to the cytoplasm of osteoblasts (ob) lining the developing bone, those being trapped in the osteoid (arrow head), and osteocytes (unfused coronal suture, #83). **G) **Corresponding phase contrast image to the central region in (F). **H) **RBP4 was not detected in fused sutures. Red blood cells had weak autofluorescence (sagittal, #5). **I-L) **GPC3 immunofluorescence (I) and H&E (J) detected protein in mesenchymal cells close to the tissue surface (arrow head) in the mid-suture region (unfused sagittal suture, #5). Membrane staining was observed for the cytoplasmic extensions of mesenchymal cells adjacent to calcified bone (K-L, unfused coronal suture, #83). **J) **H&E of section deep to (K) showing calcified bone protruding into intervening mesenchyme with osteoblasts lining the bone. Scale: 10 μm

Tissue localisation of RBP4 and GPC3 was investigated using confocal microscopy in fused and unfused tissue from coronal, sagittal, and lambdoid sutures (Fig. 9). In all unfused sutures, RBP4 was located in the cytoplasm, the most intense staining being in osteocytes in the outermost region of bone overlying the suture region on the ectocranial but not the endocranial surface (Fig. 9A, B, E). RBP4 expression was also observed in osteoblasts at the osteogenic fronts (Fig. 9C, D), those invaginating the osteoid, and by osteocytes at the bone margin (Fig. 9F, G), but not in osteocytes distal from the suture region. In the lambdoid sutures, RBP4 expression was also high in osteoblastic cells lining the cartilage fronts. No specific staining was observed in fused sutures (Fig. 9H).

Glypican 3 was most highly expressed in suture mesenchyme, particularly adjacent to the osteogenic fronts. There was distinct cell surface staining of mesenchymal cells, clearly showing the delicate branching of their cytoplasmic extensions, forming an interlacing network throughout the suture space between bone fronts (Fig. 9J–L). Osteoblasts lining osteogenic fronts and those recently invaginated also had membranous staining although considerably weaker. There was also staining of mesenchymal cells close to the tissue surface in the mid-sutural region (Fig. 9I). The higher expression in mesenchymal cells correlates with the higher mRNA and protein expression in unfused compared to fusing and fused sutures (Fig. 6).

## Discussion

Here, we have identified novel genes and attempted to gain a broader understanding of the various molecular pathways controlling suture morphogenesis in humans postnatally by analysing global gene expression differences between unfused sutures and prematurely fusing/fused sutures from patients with craniosynostosis. This is the first study in which microarray analysis has been applied to investigate differential gene expression in fused, fusing, and unfused human sutures. We identified differentially expressed genes in pathways that have been a major focus of study in craniosynostosis, including FGF, TGFβ and EGF signalling pathways. In addition, we identified genes from the Eph/ephrin pathway that has recently been linked with craniosynostosis [32,33] and the Wnt pathway that is involved osteoblast differentiation [42] and transducing FGFR signals [43]. A number of novel genes which may have important roles in suture biology and which have not been previously linked with craniosynostosis have also been identified, specifically *RBP4*, *GPC3*, and *C1QTNF3*. All three are abundantly expressed in unfused sutures and are significantly downregulated in prematurely fused sutures.

### A role for retinol binding protein 4 in regulating retinoic acid induced osteoblast differentiation

*Retinol binding protein 4 *(*RBP4*) expression was decreased 37-fold in prematurely fused/fusing sutures compared to unfused sutures by microarray analysis. RBP4 is a secretable plasma protein that classically transports retinol from liver stores to tissues where it undergoes catalytic conversion into retinoic acid (RA) [44]. Extrahepatic RBP4 expression occurs in adipose, kidney, cartilage, and brain tissue and during embryonic development of the orofacial region [45-48]. It has been speculated that tissue-specific expression of RBP4 is linked to the binding of retinol from plasma in the immediate vicinity of, and for specific use by, its tissue of origin [49,50]. Our finding of RBP4 in osteocytes on the ectocranial surface of bone directly overlying the suture mesenchyme and by cells lining the osteogenic fronts, and its dramatic downregulation in suture fusion now implicates a role for it in suture morphogenesis and craniosynostosis. At teratogenic levels, the metabolite of retinol, RA, causes craniosynostosis and other developmental anomalies [4,51,52]. At physiologic levels, however, RA represses growth of preosteoblastic cells and enhances the expression of *alkaline phosphatase*, *osteonectin*, *osteopontin*, *collagen type I *and the osteoblast specific transcription factor *RUNX2 *[53,54]. RA also stimulates *BMP2 *expression and cooperates to induce osteoblast differentiation [55]. Furthermore, primary rat calvarial osteoblasts treated with RA have increased *osteopontin *expression and switch from predominately expressing *FGFR2 *to *FGFR1*, representing a switch from active proliferation to osteoblast differentiation [56]. At physiologic levels, a function of this metabolite of retinol is therefore to suppress preosteoblastic proliferation and activate differentiation. This role of RA correlates with the localisation, reported here, of RBP4, the specific transporter of its precursor, in cells lining the ectocranial surface and the osteogenic fronts of unfused sutures and in those recently invaginated into the osteoid. RBP4 may therefore represent a primary regulator of osteogenesis in calvarial sutures by mediating the availability of retinol and its subsequent conversion to RA. In support of this, *RBP4 *knockout mice develop cranial malformations [57]. Taken together with our data on *RBP4 *expression during suture fusion and epidemiological evidence linking excess RA with craniosynostosis and other developmental anomalies [4,51,52,58] we speculate that perturbations in the RBP4-retinol-RA axis may contribute to the occurrence of craniosynostosis.

### A role for glypican 3 in maintaining suture patency

Glypican 3 (GPC3) is a cell surface heparan sulfate proteoglycan which binds to the extracellular surface of cells via a GPI (glycosyl-phosphatidyl-inisotol) anchor and is thought to facilitate interaction between various ligands and receptors. Loss-of-function mutations in *GPC3 *cause Simpson-Golabi Behmel syndrome (MIM 312870), an overgrowth syndrome with multiple skeletal abnormalities (large protruding jaw, widened nasal bridge, upturned nasal tip, and broad, short hands and fingers) that is associated with increased cell proliferation [59]. Our study shows that *GPC3 *expression is decreased 7-fold in prematurely fused/fusing sutures. GPC3 interacts with FGF2, WNT5a, and BMP-4 and -7 which are ligands of FGF receptors, WNT receptors (FZDs), and BMP receptors, respectively [60-62], all of which have been variously implicated in regulating osteoblast function. Molecular studies show that loss of GPC3 enhances the limb patterning defect of *BMP4 *heterozygous mice [62] and that GPC3 can bind FGF2 and suppress FGF2-induced cell proliferation [61]. GPC3 is also able to regulate the Wnt signalling pathway. GPC3 knockout mice exhibit an inhibition of the non-canonical Wnt/JNK signalling pathway, and activation of the canonical Wnt/β-catenin signalling pathway [63]. Canonical Wnt/β-Catenin signalling promotes osteoblast differentiation and bone accrual and inhibits osteoblast apoptosis (reviewed in [39]). We speculate that GPC3 controls cell growth within suture mesenchyme by regulating the bioavailability of FGFs, BMPs, and Wnts and might therefore acts as a gate-keeper of cell responsiveness in the suture.

### A role for C1QTNF3 in suture morphogenesis

C1QTNF3 (C1q tumor necrosis factor related protein 3), also known as CTRP3/cartducin, and CORS26, is a growth factor that hitherto had not been linked to suture morphogenesis. Our microarray study showed that *C1QTNF3 *expression is decreased 20-fold in prematurely fused/fusing sutures and qRT-PCR analysis determined that it is the short isoform of *C1QTNF3 *that is differentially expressed (Table 5, Fig. 6A). C1QTNF3 regulates proliferation of chondrocytes and their progenitor cells during both postnatal and embryonic development and has been shown to be up-regulated during BMP2 and insulin induction of chondrocyte differentiation [64]. C1QTNF3 has also been identified as promoting proliferation and migration of mouse endothelial MSS1 cells [65]. With the discovery of *C1QTNF3 *expression in unfused and fusing tissue of all sutures (Fig. 6), we predict a novel role for this growth factor in the regulation of osteoprogenitor proliferation and differentiation at the osteogenic fronts. Given our finding of cartilage-specific markers associated with posterior skull sutures we speculate that C1QTNF3 might also be involved in chondrogenesis in addition to osteogenesis in these sutures.

### The identification of cartilage in posterior sutures

Of particular interest was the identification in our microarray experiment of increased expression of genes coding for cartilage-specific *collagens types II *and *X *in the two unfused lambdoid and the unfused sagittal sutures from the posterior of the skull. The involvement of cartilage in unfused lambdoid sutures was confirmed histologically through the observation within the region of the osteogenic fronts of a cartilaginous matrix which protruded into the suture mesenchyme and adjacent calcified bone matrix (Fig. 8). In addition, confocal microscopy localised collagen type X to chondrocytes in the cartilage matrix and in osteoclasts adjacent to cartilage (Fig. 8I, J). Identification of what is termed 'secondary cartilage' has been previously noted in several human calvarial sutures, with a high incidence in normal lambdoid sutures [66,67]. It has been proposed that this secondary cartilage may develop in response to the higher mechanical forces applied to the posterior region of the skull during growth as it provides a matrix more tolerant to compression [5]. Cartilage has also been observed in rodent sutures where a cartilaginous plate underlies the lambdoid suture, possibly forming a supportive structure on which intramembranous ossification occurs [24]. Recently, cartilage and chondrocytic markers have also been identified in sagittal sutures of transgenic mice generated with the Apert syndrome FGFR2 S252W mutation [68]. The cartilage was located at the junction of the parietal and interparietal bones which corresponds to the region from which the sutures we identified as expressing cartilage-specific genes were isolated. Importantly, we demonstrated that cartilage is present within the osteogenic fronts, rather than underlying the sutures suggesting a functional rather than supportive role. Recently, *Sox9*, a regulator of chondrogenesis, has been shown to be upregulated during the initiation of posterior frontal suture closure in mice, along with the expression of *collagen types II *and *X*, followed by *collagen type I *and *osteocalcin *expression [69]. A role for endochondral ossification was therefore proposed to control fusion of this suture. Additionally, collagen types II and aggrecan have been detected in preosteogenic-condensing mesenchyme and the osteogenic fronts of developing embryonic chick heads [70]. It was proposed that, in chickens, normal intramembranous ossification includes a transient chondrogenic phase. The identification, in our study, of cartilage in lambdoid suture mesenchyme also suggests that chondrogenesis plays a role in human suture morphogenesis, particularly in the posterior skull.

### Distinctive tissue-type specific gene expression differences

The analysis of gene expression in the different suture-types indicated that gene expression profiles of unfused metopic sutures were more highly correlated with the expression exhibited by fused sutures from other suture sites; specifically they showed a significantly lower expression of those genes increased in other unfused sutures (Fig. 6A, 7). This unique expression profile may help explain the earlier occurrence of metopic suture fusion during development. Unfused coronal and unfused lambdoid sutures also showed very similar expression profiles. Both these sutures are generally formed by overlapping calvarial bone fronts, in comparison to the blunt-end sutures which form the sagittal and metopic sutures [5]. These sutures are also similar in that they are a meeting of bones of two different developmental origins (mesoderm and neural crest cells) while the sagittal and metopic sutures are the meeting point of bones of one origin, either mesoderm or neural crest, respectively.

Transcription factors are key controllers of the signalling cascades activated during development. Of those transcription factors differentially expressed between fused and unfused suture tissue a majority also showed significant expression differences between suture types. Unfused sagittal sutures had a higher expression of homeobox genes *SHOX2 *and *PAX5 *which potentially drive osteoblast differentiation and generally had increased expression in fused sutures. The unfused sagittal sutures also showed a lower expression of *MSX2*, *SIX2*, *PITX2*, *BHLH3*, and *HLF *that were generally increased in other unfused sutures compared to fused sutures. Given the higher frequency of non-syndromic craniosynostosis in the sagittal suture we speculate that a lower expression of transcription factors associated with unfused sutures, and a higher expression of those associated with fused sutures, might leave this suture more vulnerable to premature closure. Both unfused sagittal and unfused coronal sutures had significantly higher expression of members of the FOS (*FOS *and *FOSB*) and JUN (*JUN *and *JUNB*) oncogene families compared to unfused lambdoid sutures, particularly for coronal sutures (eg. *FOSB *was 54.4-fold increased in the coronal sutures, compared to 20.2-fold in the sagittal sutures). Through homo- and hetero-dimerisation, these proteins form the AP-1 transcription complex. Increased *Jun *and *Fos *expression occurs in prematurely fused mouse sutures induced by the application of FGF2-soaked beads [71]. It was suggested that FGF signalling increases expression of the AP-1 complex which then induces expression of osteopontin and osteoblast differentiation, resulting in premature suture closure. It is also known that the coronal and sagittal sutures are those most frequently affected in FGFR syndromic craniosynostoses. We therefore suggest that the higher expression of AP-1 transcription factor components in coronal and sagittal sutures makes them more responsive to the increased or inappropriate FGF signalling caused by gain-of-function *FGFR *mutations. However, it was also observed that unfused sagittal sutures had decreased expression of *FGFR2 *compared to the coronal suture. This may explain why mutations in these genes most commonly affect the coronal suture rather than the sagittal suture.

Another important finding from our microarray analysis was that there was limited difference in whole genome expression between non-syndromic and syndromic patient samples. This was verified by qRT-PCR using 35 samples from 7 syndromic and 11 non-syndromic patients, analysing 11 genes differentially expressed between unfused and fused sutures. We note, however, that these genes were identified as being significantly differentially expressed during premature fusion by using tissue groups which contained samples from patients with different aetiologies. Thus, we specifically identified genes which were not specific to one aetiology. If these two groups of samples were analysed independently, it is likely that there may be a small proportion of genes which are differentially expressed between samples from patients with different aetiologies and these genes will be directly related to the mutation of initiation. However, in this study we were not so interested in these aetiology specific indicators, but rather the general mechanisms underlying craniosynostosis. Significantly, the results from our microarray hierarchical analysis study indicate that the genes involved in the pathogenesis of different types of craniosynostosis are more similar than may previously have been thought.

## Conclusion

Through the analysis of human suture material we have identified a large number of novel differentially expressed genes, three of which, *RBP4*, *GPC3 *and *C1QTNF3*, we believe may have significant regulatory roles in the control of both suture patency and growth. Furthermore, we have identified significant gene expression differences between human sutures from different cranial sites and identified the involvement of cartilage in posterior calvarial sutures, particularly the lambdoid suture. These data open up new avenues of investigation in respect to the molecular mechanisms underlying the different responses of calvarial sutures to mutations causing craniosynostosis. This information is vital for the development of therapeutic agents to control skull growth in children with sutural defects, as well as providing clinicians with a better understanding of the developmental mechanisms operating in different sutures.

## Methods

### Tissue Samples

Calvarial suture samples were obtained from patients undergoing transcranial surgery for syndromic or non-syndromic craniosynostosis. Patients were genotyped for all known *FGFR1*-3 and *TWIST1 *mutations (Table 1) [72]. Samples used for microarray analysis (n = 16) were taken from males (n = 5) aged 3-7 months. Additional samples used for validation experiments (n = 25) and histology (n = 9) were taken from female (n = 8) and male (n = 10) patients aged 3-40 months. Additionally, six unfused suture samples were obtained from patients aged 1 day to 91 months undergoing transcranial surgery for reasons other than craniosynostosis (Table 1). Consent was provided by all guardians in line with the guidelines of the Research Ethics Committee of the Children, Youth and Women's Health Service, Adelaide, South Australia. Suture tissue was taken from prematurely fused/fusing and/or patent sutures from one or more of the sagittal, coronal, lambdoid, and metopic sutures. The site and fusion state of samples used for each analysis type are indicated in Table 1. Specimens used for microarray analysis and validation experiments were stored in RNAlater (Ambion, Austin, TX, USA) at -20°C. Specimens used for histology and immunofluorescence were fixed in formalin, decalcified with 10% EDTA, pH 7.4, by standard procedures and stored in 100% ethanol.

### 3DCT and MicroCT scans

Stage of fusion was confirmed by assessing 3D computer tomography (CT) images taken prior to surgery. Selected suture samples underwent MicroCT analysis to determine the degree of fusion, as previously described [73]. Briefly, tissues samples were placed in RNAlater, enclosed tightly in an acrylic tube, and analysed with a SkyScan 1072 MicroCT scanner (SkyScan, Antwerp, Belgium). 2D images were used to generate 3D reconstructions using 3D creator software (SkySkan).

### Total RNA isolation

Tissues used for microarray analysis and validation experiments had the suture proper (suture mesenchyme + 3 mm bone on either side for unfused sutures, or fused bony ridge + 3 mm bone on either side for fused sutures) dissected from all specimens and the overlying pericranium was removed. Tissue samples were cut into 30-40 μg pieces for RNA extraction, snap frozen, crushed between cryogenically cooled steel blocks, and homogenised in 2 ml TRIreagent (Molecular Research Center, Cincinnati, OH, USA) using a Mini-Bead-Beater-8 (BioSpec Products, Bartlesville, OK, USA). RNA was isolated from supernatant following recommendations by Naderi *et al*. [74]. Briefly, separated aqueous phase was twice extracted with chloroform and precipitated with 1 volume isopropanol, 0.1 volume 7.5 M ammonium acetate, and 5 μg/ml linear polyacrylamide (Ambion) at -20°C overnight. Pelleted RNA was washed twice with 70% ethanol and resuspended in RNA Storage Solution (Ambion). RNA extracts from the same sample were combined. 10 μg of each combined RNA sample was purified and concentrated to greater than 300 ng/μl with phenol:chloroform:isoamyl alcohol (25:24:1) extraction. Total RNA quality was determined by analysing the integrity of the 28S and 18S ribosomal bands on a non-denaturing 1.5% agarose Tris-borate buffered gel and determining RNA purity by A260:280 ratios using UV spectroscopy.

### Microarray cDNA synthesis, hybridisation, and scanning

RNA from 16 tissue samples (Table 1) was analysed using the Affymetrix expression microarray Human U133A 2.0 GeneChip platform. Concentrated total RNA was prepared for hybridisation to the GeneChips following a one-cycle target labelling protocol (Affymetrix GeneChip Expression Analysis Technical Manual). RNA was reverse transcribed into double stranded cDNA using SuperScript II (Invitrogen, Gaithersburg, MD, USA) with T7-oligomers. Poly-A RNA spike-in controls were added along with 2 μg of total RNA to all cDNA reactions. Biotin labelled cRNA was prepared from the cDNA using the GeneChip IVT labelling system (Affymetrix, Santa Clara, CA, USA), with incubation at 37°C for 16 hours. 10 μg of fragmented cRNA was hybridised to each Affymetrix U133A 2.0 GeneChip. Array hybridisation, staining, and washing was carried out following manufacture's protocols using the Fluidics Station 400 (Affymetrix). Stained arrays were scanned on a GeneChip Scanner 3000 (Affymetrix) controlled by GCOS software (Affymetrix).

### Microarray data analysis

CEL files containing probe intensity data were analysed in R using Bioconductor packages [75,76]. Quality control analyses were carried out on probe level model (PLM) normalised samples. Normalised un-scaled standard error (NUSE) box plots, Mbox plots, and RNA degradation plots were analysed [77]. For statistical analyses probe intensity data were normalised using the GeneChip Robust Multichip Average (GCRMA) algorithm, which has been shown to provide a good balance between accuracy and precision [78]. Diana-divisive hierarchal clustering was used to cluster the microarray samples based on whole genome expression values. To identify differential gene expression the Limma package was used to fit linear models to the data, incorporating an empirical Bayes modification of the standard errors [79]. False discovery rate adjustment of P-values was performed to account for multiple testing [80]. Correlation plots and hierarchal trees were generated using cluster packages available in R. Gene ontology over-representation was analysed using GOTree Machine, normalising to the U133A 2.0 gene set, with significance set at *P *< 0.01 [81,82]. Gene set enrichment analysis was carried out using GSEA v 2.01, comparing the ranked list of 2-fold differentially expressed probe sets to gene sets c1-c4 (v2.symbols.gmt) [83,84]. Gene set exclusion was set at min = 4, max = 500, with 1000 weighted permutations executed. A 3-way contrast matrix was created for the unfused suture comparisons: coronal-sagittal, lambdoid-sagittal, and lambdoid-coronal. Using the Limma package a Venn diagram was produced for those genes identified to be differentially expressed using a nested F-test approach which gives particular attention to genes which are differentially expressed (*P *< 0.01) under 2 or more conditions. To control for patient-specific effects due to the small sample size of this comparison, we performed a set of separate pair wise comparisons using a matched pairs design. In this case we first restricted analysis to patients with a sample from each of the sutures of interest, and then performed analysis on the within patient differences. A matched-pairs matrix was constructed for an unfused coronal-lambdoid comparison using samples from patients #36 and #46 and a coronal and sagittal comparison using samples from patients #36 and #58. A t-test was performed between unfused lambdoid and sagittal samples from patients #36, #46 and #58. A linear model incorporating an empirical Bayes modification was applied to each matched pairs comparison. To identify genes differentially expressed in unfused sagittal sutures, the intersection of the 3-way and matched pairs coronal-sagittal comparison and lambdoid-sagittal comparisons was found.

### Realtime quantitative RT-PCR (qRT-PCR)

Total RNA was reverse transcribed into cDNA using SuperScript III (Invitrogen). Two micrograms of RNA was added to 40 μl total volume reactions which were carried out following the manufacturer's protocol. In addition to the patient RNA samples, a calibrator RNA sample which was used to standardise absolute qRT-PCR results, was transcribed into cDNA, column purified (QIAquick PCR purification kit, Qiagen, Clifton Hill, VIC, Australia) and quantified by UV spectroscopy. cDNA from all samples was diluted 1/3 and 1/120 with TE (pH 8) and supplemented with herring sperm DNA to 1 ng/μl. The 1/120 dilutions were used for the amplification of the 18S rRNA gene and the 1/3 dilutions were used for the analysis of all other genes. Absolute quantification was carried out using standard curves generated by serial dilution of target amplicon-containing plasmids (pGEM-T easy, Promega, Annandale, NSW, Australia), to cover up to 5 logs of amplicon copy number per microlitre. All primers were designed to target the same sequence as the microarray probes, to overlap exon-exon junctions, and to have a melting temperature of 60°C (Additional file 13). Realtime reactions were carried out using SYBR green (Applied Biosystems, Foster City, CA, USA) on a ABI Prism 7000 Sequence Detection System (Applied Biosystems). Twenty microlitre reactions contained 2 μl of cDNA and 0.4 μM each primer. PCR amplification followed a two step cycling protocol; 10 min denaturation at 95°C followed by 40 cycles of 95°C for 15 s and 60°C for 1 min. Melting curve analysis was conducted to confirm specific amplicon amplification. Patient reactions were performed in triplicate and standard curve points in duplicate. ABI Sequence Detection Software version 1.2 was used to determine sample Ct values, with the same threshold set for all reactions. Absolute copy number values calculated from standard curves were normalised to a calibrator cDNA sample (1 ng was used for RT-PCR) by calculating a ratio of the patient 18S Ct to that of the calibrator sample 18S Ct. Differences between samples were analysed for log_10 _transformed data by Student's t-test, with significance set at *P *< 0.05.

### Western blot analysis

Whole tissue protein was isolated from the TRIreagent organic phase separated during RNA extraction following the manufacturer's instructions and reconstituting in 10 M urea. Protein was quantified using the Bio-Rad Protein Assay (Bio-Rad, Regent Park, NSW, Australia) and 25 ng of total protein was resolved by 10% SDS-PAGE and transferred to a Hybond-C nitrocellulose membrane (Ammersham, North. Ryde, NSW, Australia). Membranes were blocked with Odyssey Blocking Buffer (LI-COR Biosciences, Lincoln, NE, USA) diluted 1:1 in TBS (50 mM TRIS pH 7.5, 150 mM NaCl) for 45 min. Antibodies were diluted 1:1 in blocking buffer with 0.1% Tween-20. Blocked membranes were probed with either mouse monoclonal anti-collagen type I (COL1, 1:100, Calbiochem, Alexandria, NSW, Australia) or goat anti-mouse CORS26/C1qTNF3 (1:50, R&D Systems, Minneapolis, MN, USA) and incubated overnight at 4°C. COL1-probed membranes were double probed with rabbit polyclonal anti-human retinol-binding protein (RBP4, 1:20000, DAKO, Botany, NSW, Australia) for 1 h at room temperature (RT). Membranes were washed three times in TBST (50 mM TRIS pH 7.5, 150 mM NaCl, 0.1% Tween-20) following primary antibody incubation. Antibody binding to double probed membranes was detected by infrared emission using goat anti-rabbit Alexa Flour 680 (1/20000, Molecular probes, Eugene, OR, USA) and goat anti-mouse IRDye 800 (1:15000, Rockland, Gilbertsville, PA, USA). Antibody binding to single probed membranes was detected with donkey anti-goat Alexa Fluor 680 (1:10000, Molecular Probes). Protein bands were detected and quantified using the Odyssey infrared imaging system (LI-COR Biosciences). Double probed membranes were stripped with low pH stripping buffer (25 mM glycine-HCL pH 2, 1% (w/v) SDS) for 30 min at RT, followed by washing in TBST. Stripped membranes were blocked in 5% skimmed milk in TBST for 30 min. Membranes were probed with primary sheep anti-human glypican 3 (GPC3, 1:2000, R&D Systems), followed by rabbit anti-sheep horseradish peroxidase-conjugated antibody (1:2000, Chemicon) and Immobilon Western Substrate (Millipore, North Ryde NSW, Australia). Antibodies for GPC3 detection were diluted in 5% skimmed milk in TBST and incubated for 1 h at RT. For densitometry, GPC3 blots were scanned using the Odyssey imaging system (LI-COR Biosciences).

### Immunofluorescence confocal microscopy

Fixed and decalcified specimens were dehydrated through a graded ethanol series and embedded in paraffin. Sections were cut to 3 μm thickness, mounted on 3-aminotriethoxysilane (APES)-coated slides and incubated for 16 h at 60°C, followed by 7 h at 37°C. Sections were deparaffinised and rehydrated in distilled H_2_O for 5 min. Antigen retrieval was carried out using TEG buffer (TRIS-EGTA, pH 9.0) for RBP4 and COLX and TRIS-HCl buffer (pH 1.0) for GPC3. All sections were incubated at 60-70°C overnight with constant stirring. Slides were cooled and washed in 1 × PBS (pH 7.4) before incubating in blocking buffer (0.3% casein, 0.1% Tween-20, in 1 × PBS pH 7.4) for 15 min. Rabbit polyclonal anti-human RBP4 (1:2000, DAKO), sheep anti-human GPC3 (1:50, R&D Systems), and mouse monoclonal anti-collagen type X (COLX, 1:500, Sigma-Aldrich, Castle Hill, NSW, Australia) primary antibodies were incubated for 1 h, followed by washing in 1 × PBS and Tween 20 (0.1%). Sections were incubated with corresponding secondary antibodies (goat anti-rabbit Alexa Fluor 488, donkey anti-sheep Alexa Fluor 488, goat anti-mouse Alexa Flour 488) for 1 h at RT followed by washing. All antibodies were diluted in 1 × PBS. Sections were coverslipped using ProLong Gold antifade (Invitrogen) and viewed with a Leica TCS 4D confocal laser scanning microscope (Leica Laser Technology, Heidelberg, Germany). Secondary antibodies was excitated with a 488-nm laser and fluorescent light detected using a FITC band pass 520-560 nm barrier filter. The controls were prepared in the absence of the primary and the secondary antibodies, and with both antibodies but without antigen retrieval, and were negative in all cases.

### Histology and cartilage detection

Decalcified formalin fixed specimens were sectioned (3 μm), mounted onto APES-coated slides, and incubated at 60°C for 16 h. Deparaffinised and rehydrated sections were stained with 1% Alcian blue in 3% aqueous acetic acid (pH 2.5) to detect the presence of cartilage, or hematoxylin and eosin for tissue structure, and mounted in Depex (Sigma-Aldrich). Sections were imaged using a brightfield microscope (Carl Zeiss Jena, Jena, Germany) equipped with a DFC480 digital camera (Leica Microsystems).

## Authors' contributions

AKC performed RNA and protein isolation and quantification, RNA processing and microarray scanning and data mining, realtime quantitative RT-PCR and Western blot validation experiments, confocal immunofluorescence and histological analysis of suture tissues, and drafted the paper. Immunofluorescence and histological stainings were reviewed and discussed by AKC, IH, PA, and BP. PJA collected the suture samples and performed decalcification and fixation of specimens. CRW coordinated microarray quality control, data mining, and statistical analysis of quantitative RT-PCR validation experiments. BP assisted with drafting of the paper. AKC, AVD, BP, PA, PM, and IH participated in study's design and coordination. All authors have reviewed the manuscript. BP, IH, and AKC were the final editors of the manuscript.

## Supplementary Material

Additional file 1 Microarray quality control RNA digestion plot. RNA digestion plot, for the 16 RNA samples hybridised to Affymetrix Human expression U133A 2.0 GeneChip microarrays. RNA digestion plot compares expression intensity for all probes which bind sequences in order of 5' to 3' along a transcript, for all probe sets on the GeneChip. Click here for file

Additional file 2 Microarray quality control NUSE box plots. Normalised unscaled standard error (NUSE) box blots for the 16 RNA samples hybridised to Affymetrix Human expression U133A 2.0 GeneChip microarrays. NUSE box plot shows a ratio of the NUSE for each probe set compared to a median value of the NUSEs across all arrays. Click here for file

Additional file 3 Microarray quality control Mbox plots. Mbox plots for the 16 RNA samples hybridised to Affymetrix Human expression U133A 2.0 GeneChip microarrays. M box plot shows the range of fold change (M) for each probe set expressed by one sample compared to the mean expression of that probe set across all samples analysed. Click here for file

Additional file 4 Hierarchical cluster based on whole genome expression. Diana divisive hierarchical cluster of the 16 tissue samples analysed based on the expression intensity of all probe sets on the microarray. Click here for file

Additional file 5 Genes with > 2-fold differential expression between sutures. List of the top 829 differentially expressed genes with > 2-fold differential expression between fusing/fused and unfused suture tissue. A positive value indicates higher levels in fused sutures and a negative value indicates higher expression in unfused sutures. Click here for file

Additional file 6 Enrichments plots generated by GSEA analysis for selected significantly correlated datasets. The colour bar depicts phenotype correlation based on ranking metric scores. Red indicates those genes with increased expression in fused sutures and blue indicates those genes with increased expression in unfused sutures. Black bars represent genes ordered by their ranking within the 2-fold differentially expressed gene list between fused/fusing and unfused sutures. Click here for file

Additional file 7 H&E analysis of suture tissue. A large number of white blood cells were observed in the calvarial tissue. The majority of cells appear as lymphocytes (*). Click here for file

Additional file 8 Genes identified to be differentially expressed (*P *< 0.01) by matched pairs analysis between unfused coronal and sagittal sutures. A positive value indicates higher expression levels in coronal sutures and a negative value indicates higher expression in sagittal sutures. Click here for file

Additional file 9 Genes identified to be differentially expressed (*P *< 0.01) by matched pairs analysis between unfused lambdoid and sagittal sutures. A positive value indicates higher expression levels in lambdoid sutures and a negative value indicates higher expression in sagittal sutures. Click here for file

Additional file 10 Genes identified to be differentially expressed (*P *< 0.01) by matched pairs analysis between unfused coronal and lambdoid sutures. A positive value indicates higher expression levels in coronal sutures and a negative value indicates higher expression in lambdoid sutures. Click here for file

Additional file 11 Syndromic and non-syndromic sutures are not significantly different. P-values from Student's t-test comparing gene expression between syndromic and non-syndromic samples from for each stage of development. Samples from different suture sites were combined for analysis. Click here for file

Additional file 12 qRT-PCR results for the additional genes not shown in Figure 6A. The long isoform of *C1QTNF3 *had limited to no differential expression between unfused, fusing, and fused suture from the different sites and was therefore not the highly significantly differentially expressed isoform. *COL8A2 *and *COL3A1 *had increased expression in unfused compared to fused sutures, except in the sagittal suture. Click here for file

Additional file 13 Primers used for realtime qRT-PCR. Click here for file
