# Supplementary figures and images for: Unravelling the molecular control of calvarial suture fusion in children with craniosynostosis
Source: BMC Genomics. 2007 Dec 12;8:458. doi: 10.1186/1471-2164-8-458 (PMC2222648; doi:10.1186/1471-2164-8-458)

RNA digestion plot

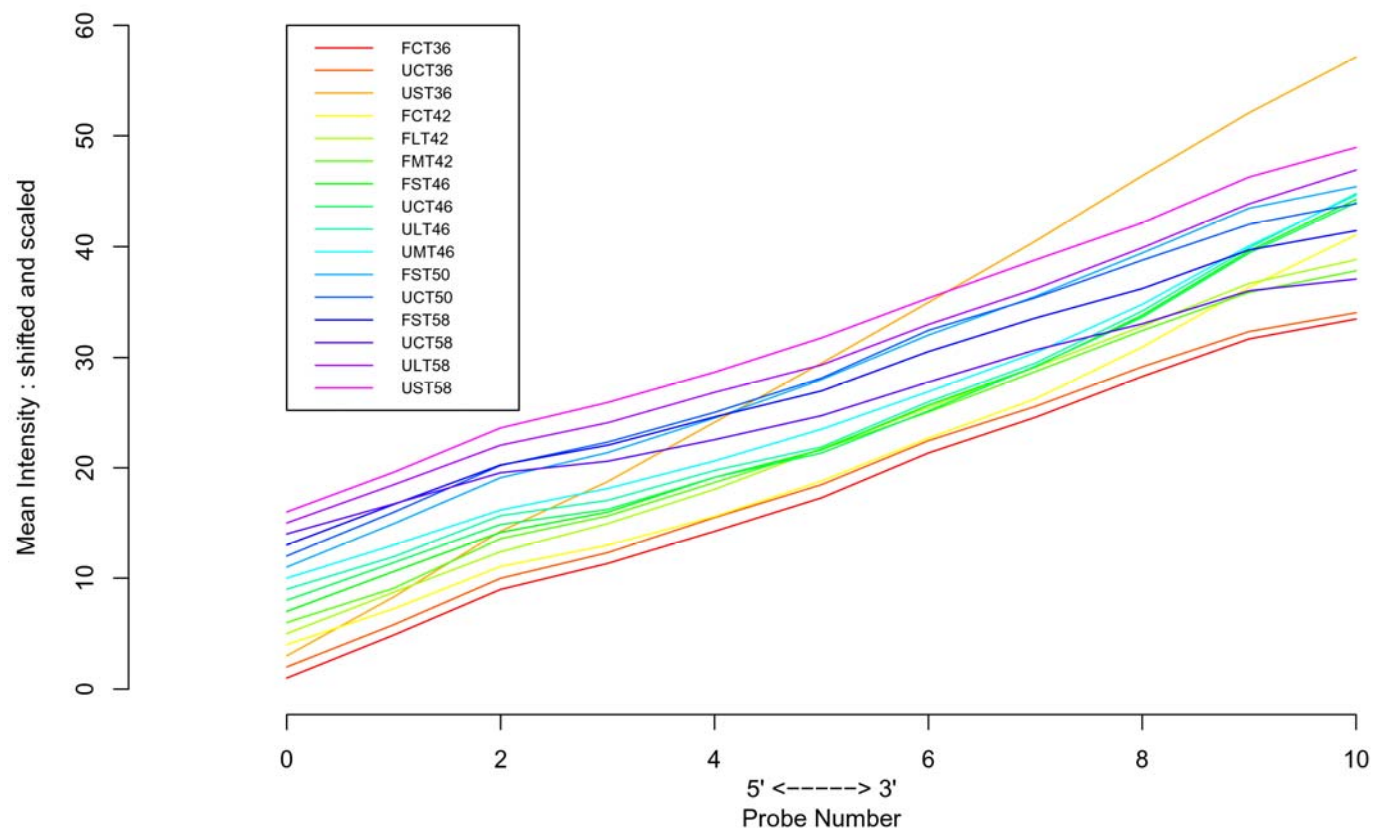

Supplement: Additional file 1 — Microarray quality control RNA digestion plot. RNA digestion plot, for the 16 RNA samples hybridised to Affymetrix Human expression U133A 2.0 GeneChip microarrays. RNA digestion plot compares expression intensity for all probes which bind sequences in order of 5' to 3' along a transcript, for all probe sets on the GeneChip. [file 1471-2164-8-458-S1.pdf]

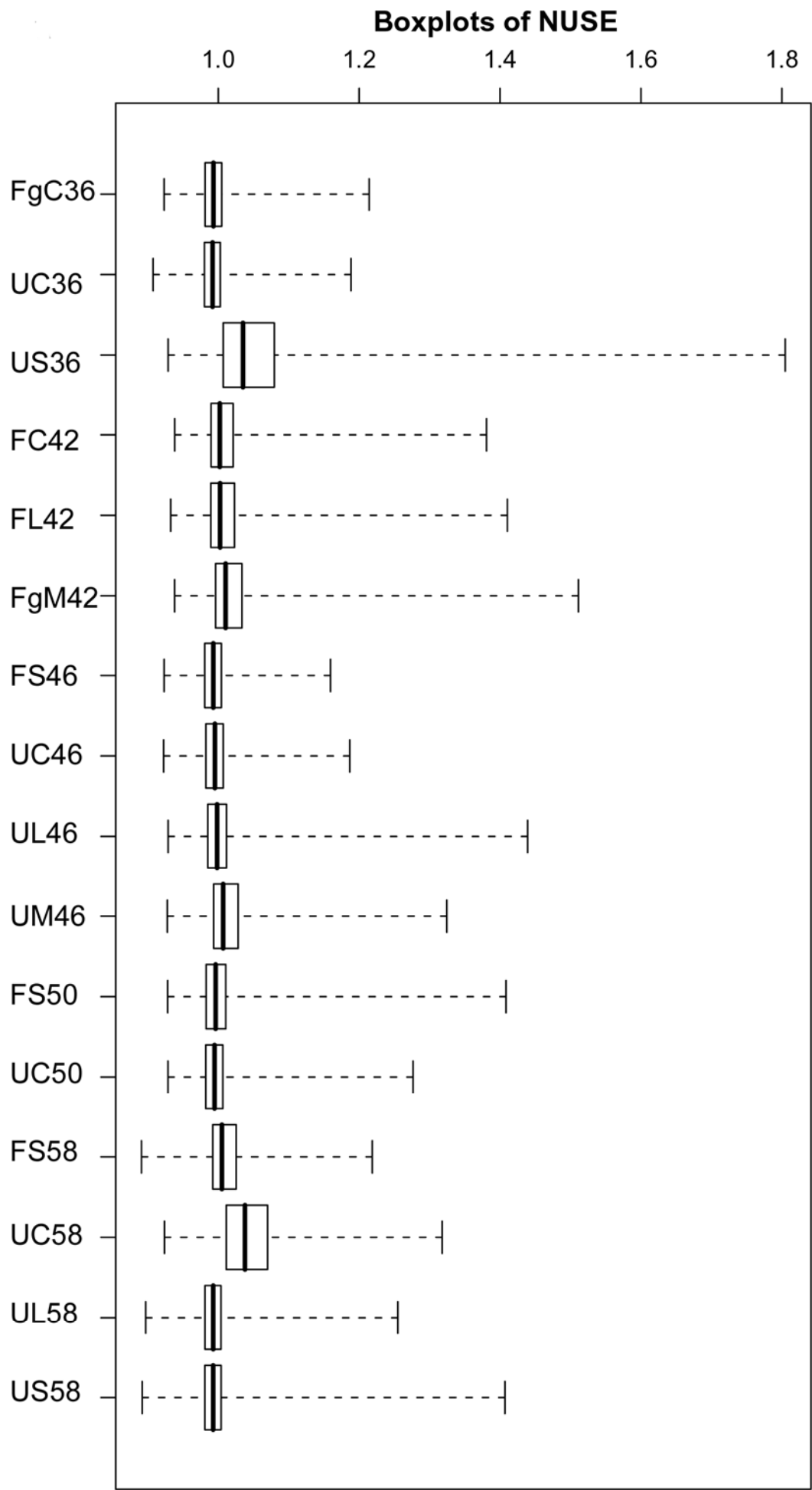

Supplement: Additional file 2 — Microarray quality control NUSE box plots. Normalised unscaled standard error (NUSE) box blots for the 16 RNA samples hybridised to Affymetrix Human expression U133A 2.0 GeneChip microarrays. NUSE box plot shows a ratio of the NUSE for each probe set compared to a median value of the NUSEs across all arrays. [file 1471-2164-8-458-S2.pdf]

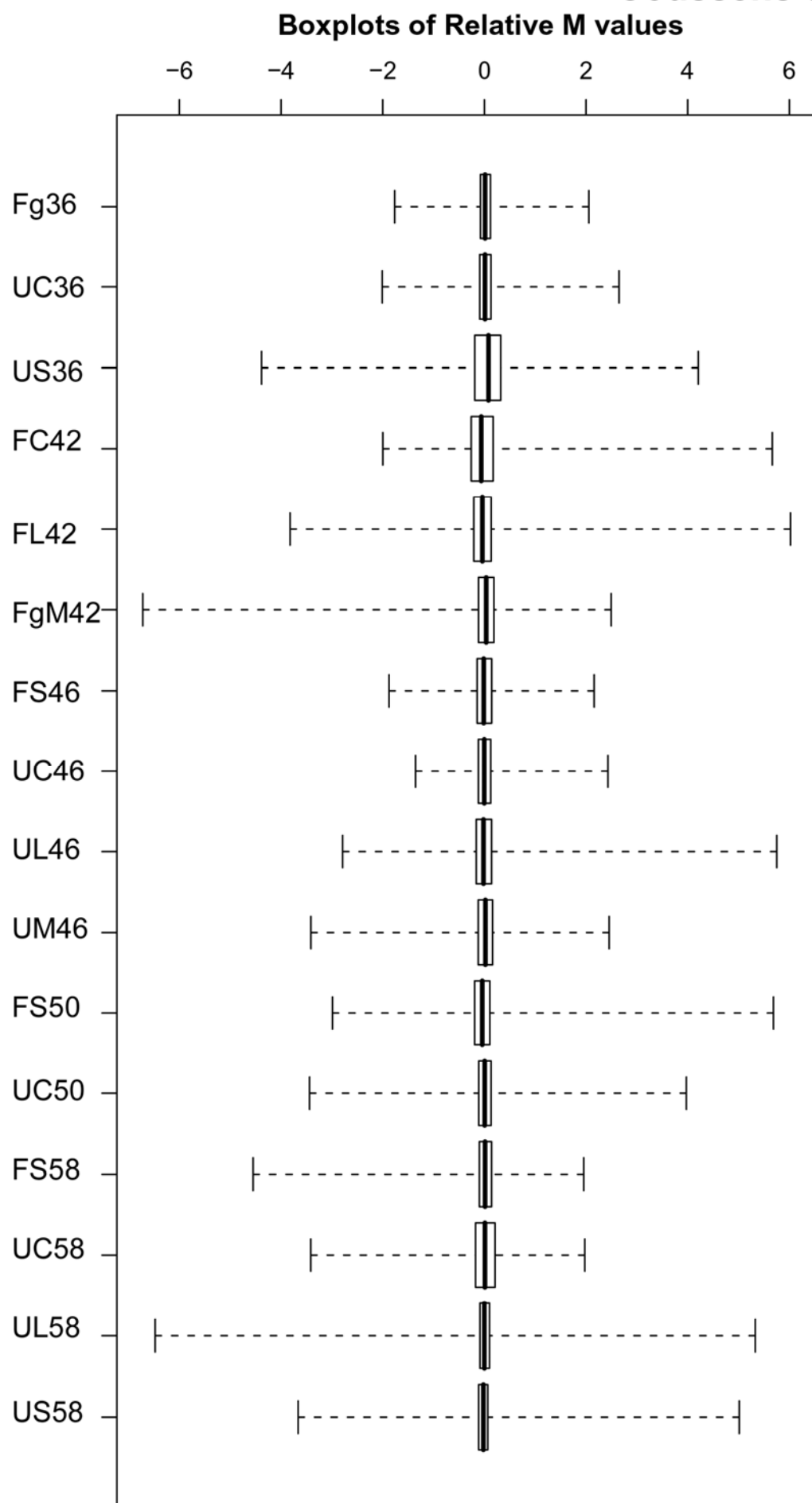

Supplement: Additional file 3 — Microarray quality control Mbox plots. Mbox plots for the 16 RNA samples hybridised to Affymetrix Human expression U133A 2.0 GeneChip microarrays. M box plot shows the range of fold change (M) for each probe set expressed by one sample compared to the mean expression of that probe set across all samples analysed. [file 1471-2164-8-458-S3.pdf]

## Additional file 4

Coussens et al.

### Hierarchical Cluster Based on All Genes

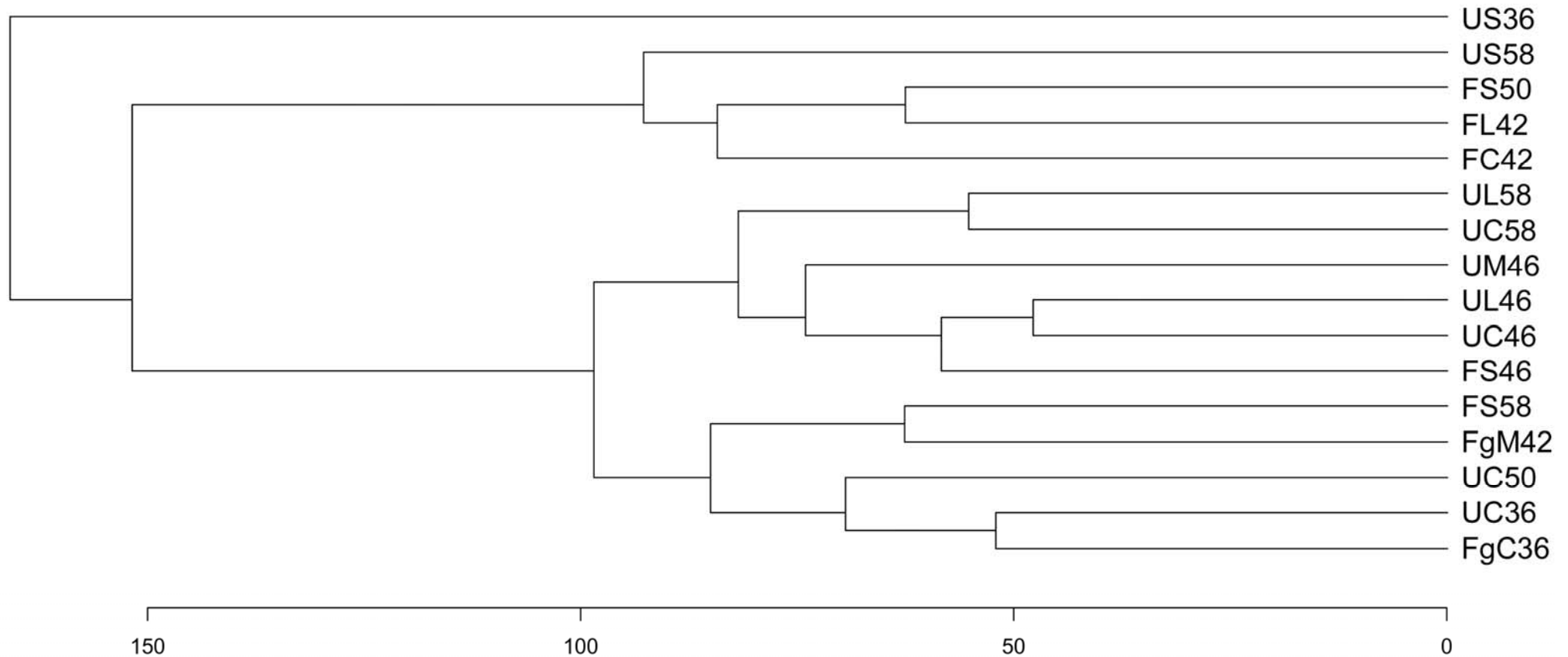

Supplement: Additional file 4 — Hierarchical cluster based on whole genome expression. Diana divisive hierarchical cluster of the 16 tissue samples analysed based on the expression intensity of all probe sets on the microarray. [file 1471-2164-8-458-S4.pdf]

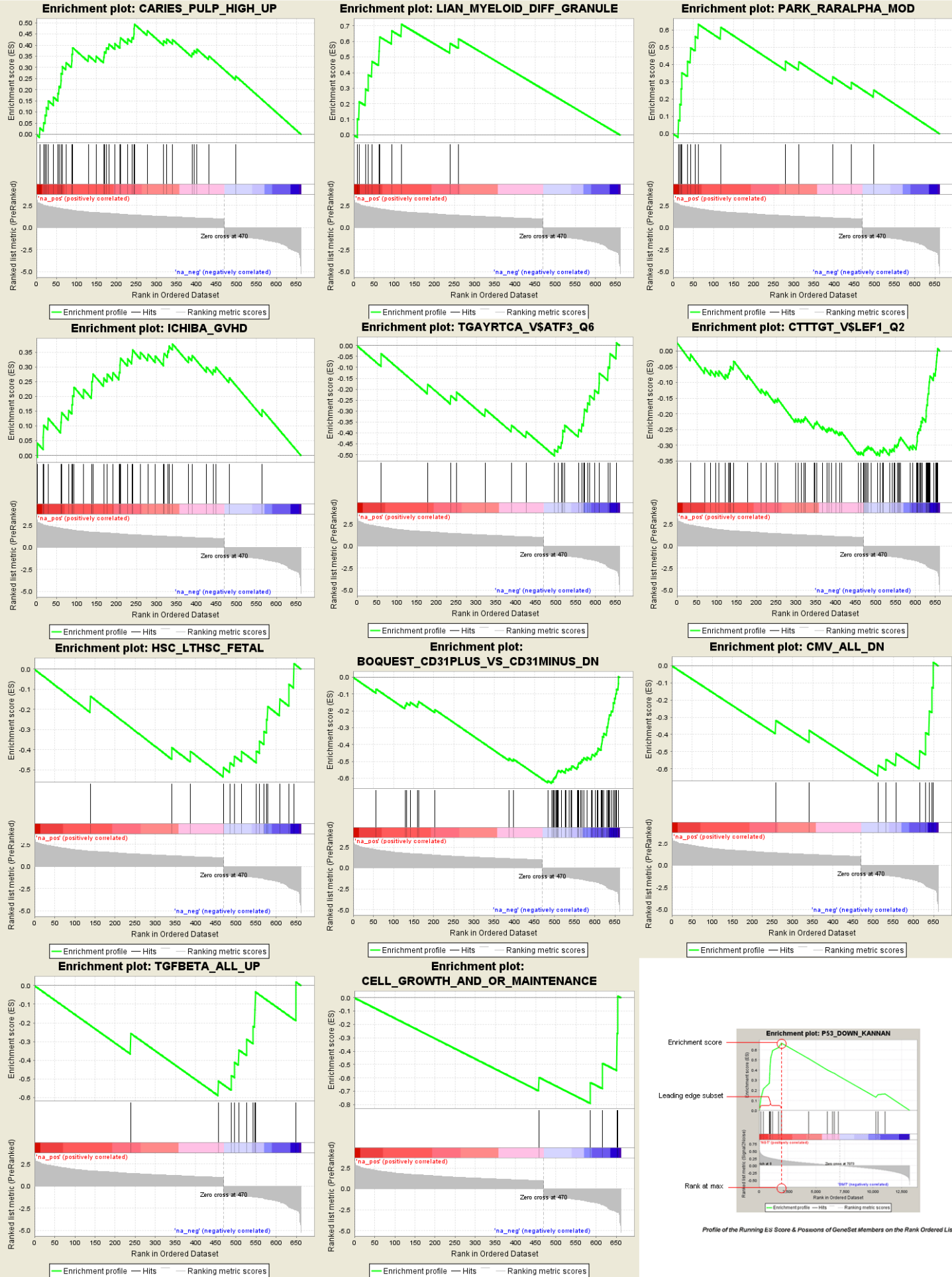

Supplement: Additional file 6 — Enrichments plots generated by GSEA analysis for selected significantly correlated datasets. The colour bar depicts phenotype correlation based on ranking metric scores. Red indicates those genes with increased expression in fused sutures and blue indicates those genes with increased expression in unfused sutures. Black bars represent genes ordered by their ranking within the 2-fold differentially expressed gene list between fused/fusing and unfused sutures. [file 1471-2164-8-458-S6.pdf]
