# Supplementary material for: Unravelling the molecular control of calvarial suture fusion in children with craniosynostosis
Source: BMC Genomics. 2007 Dec 12;8:458. doi: 10.1186/1471-2164-8-458 (PMC2222648; doi:10.1186/1471-2164-8-458)
Supplement: Additional file 7 — H&E analysis of suture tissue. A large number of white blood cells were observed in the calvarial tissue. The majority of cells appear as lymphocytes (*). [file 1471-2164-8-458-S7.pdf]

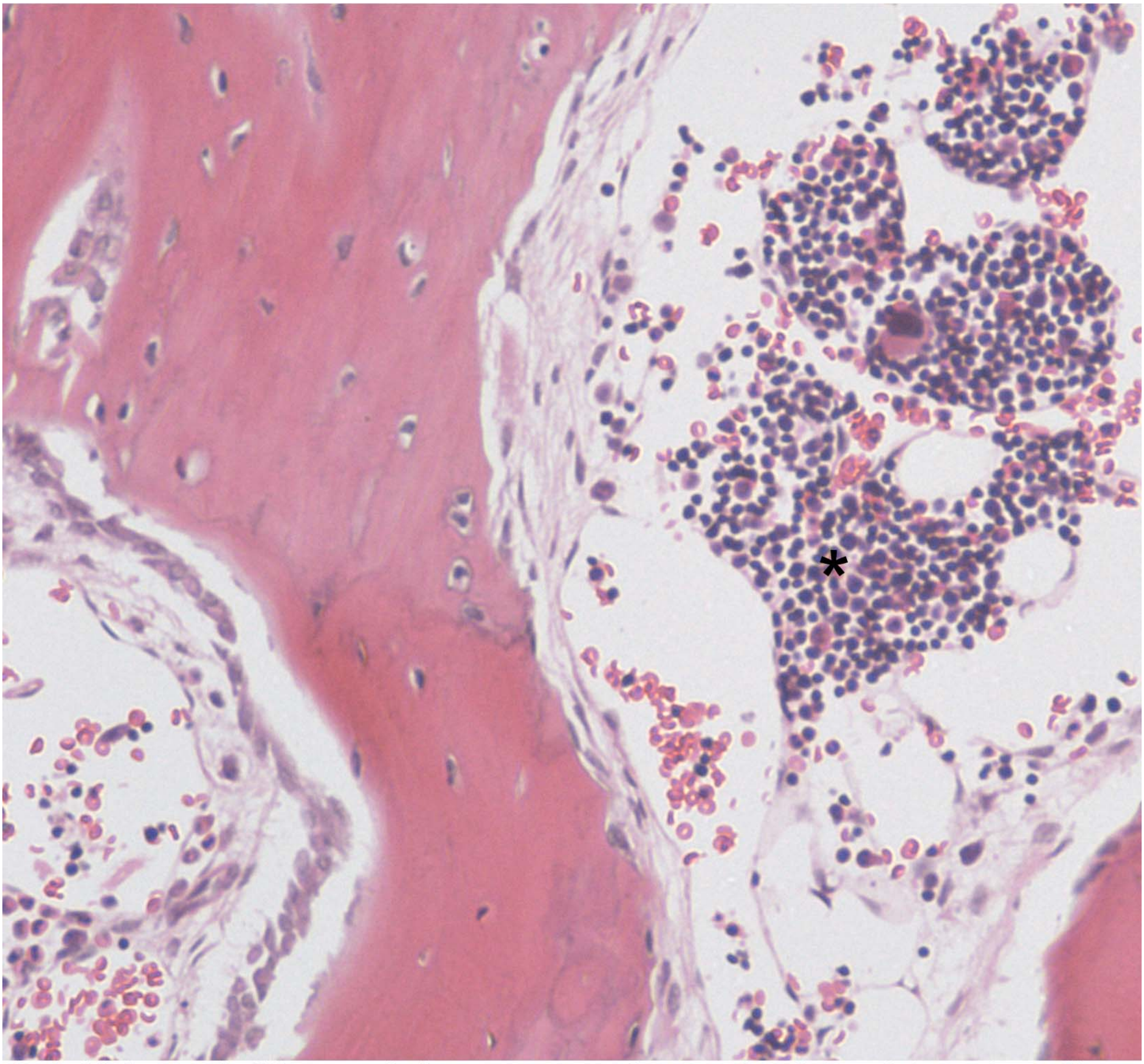

Additional file 7. A large number of white blood cells were observed in the calvarial tissue . The majority of cells appear as lymphocytes (*).
