# Supplementary material for: Unravelling the molecular control of calvarial suture fusion in children with craniosynostosis
Source: BMC Genomics. 2007 Dec 12;8:458. doi: 10.1186/1471-2164-8-458 (PMC2222648; doi:10.1186/1471-2164-8-458)
Supplement: Additional file 12 — qRT-PCR results for the additional genes not shown in Figure 6A. The long isoform of C1QTNF3 had limited to no differential expression between unfused, fusing, and fused suture from the different sites and was therefore not the highly significantly differentially expressed isoform. COL8A2 and COL3A1 had increased expression in unfused compared to fused sutures, except in the sagittal suture. [file 1471-2164-8-458-S12.pdf]

Coussens et al.

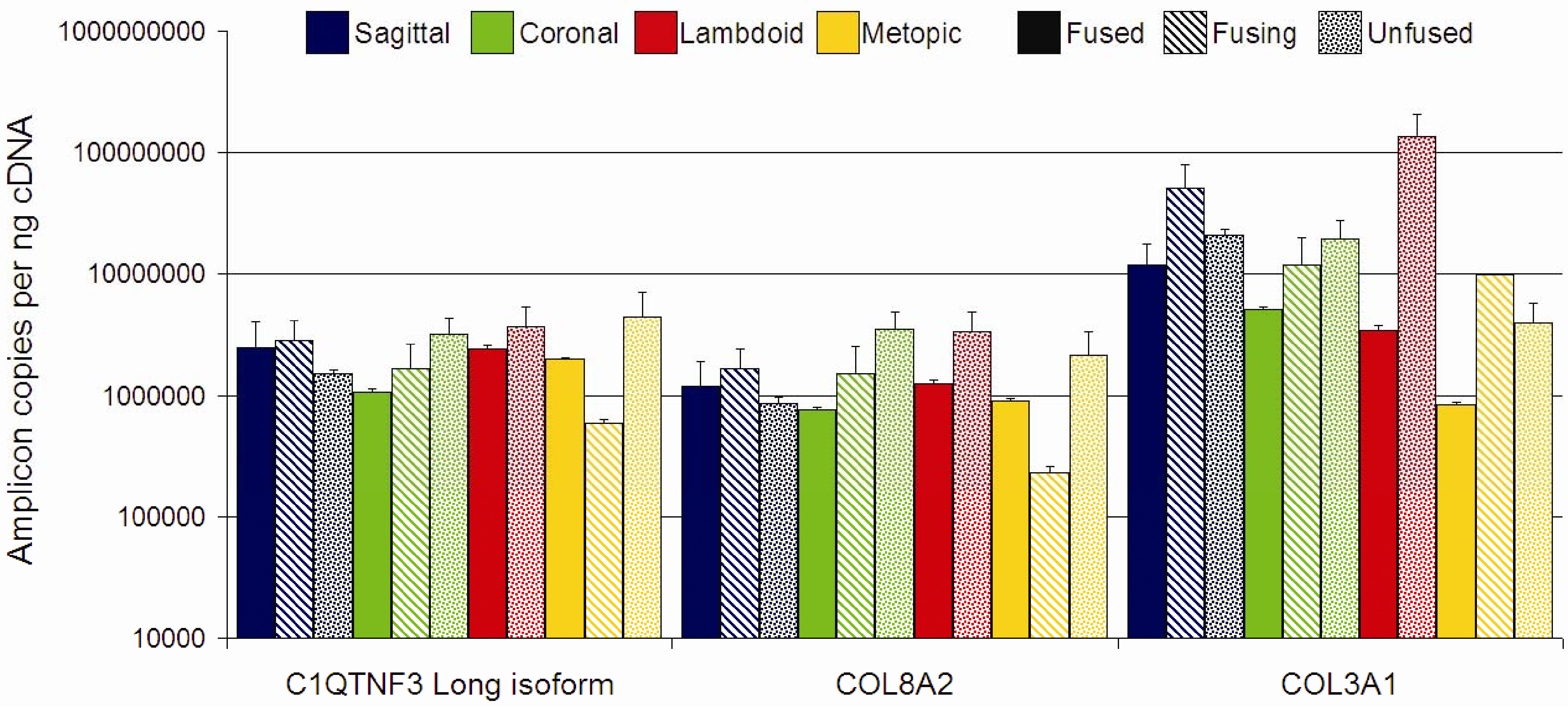

Additional file 12. qRT-PCR results for the additional genes not shown in Figure 6A. The long isoform of *C1QTNF3* had limited to no differential expression between unfused, fusing, and fused suture from the different sites and was therefore not the highly significantly differentially expressed isoform. *COL8A2* and *COL3A1* had increased expression in unfused compared to fused sutures, except in the sagittal suture.
